# Pore-Size-Dependent Competitive CH_4_/CO_2_ Adsorption in HRTEM-Informed Short-Chain-Aromatic Organic-Matter Nanopores of Coal-Measure Shale

**DOI:** 10.3390/molecules31214038

**Published:** 2026-11-09

**Authors:** Miao Long, Kunjie Li, Peng Xia, Yangyang Li, Jie Wu

**Affiliations:** 1 Yi’an Lanyan Coal and Coalbed Methane Co-Mining Technology Co., Ltd., Taiyuan 030006, China; t19135501952@163.com (Y.L.); 15235606904@163.com (J.W.); 2 College of Resources and Environmental Engineering, Guizhou University, Guiyang 550025, China; 19808682458@163.com (M.L.); pxia@gzu.edu.cn (P.X.); 3 State Key Laboratory of Coal and CBM Co-Mining, Taiyuan 030006, China

**Keywords:** lattice-fringe statistics, ring-cluster distribution, grand canonical Monte Carlo, binary-mixture selectivity, nanoconfinement

## Abstract

This study investigates pore-size-dependent competitive CH_4_/CO_2_ adsorption in coal-measure shale organic-matter nanopores represented by HRTEM-informed short-chain aromatic fragments. Coal-measure shale from the eastern Yushe Block of the Qinshui Basin was selected as the research object. High-resolution transmission electron microscopy was employed to quantitatively characterize the aromatic lamellar structure of the organic matter. Based on the obtained structural parameters, organic-matter pore models with pore sizes ranging from 1 to 4 nm were constructed. Grand canonical Monte Carlo simulations were subsequently performed to investigate the single-component and competitive adsorption behaviors of CH_4_ and CO_2_ at temperatures of 288–308 K and pressures of 0.25–8 MPa. The results indicate that the aromatic structure of the organic matter is characterized by short chains and a low degree of condensation, while the pore system is dominated by micropores and small mesopores. CH_4_ exhibited its highest nominal-pore-volume-normalized adsorption in the 1 nm pore, consistent with micropore filling, whereas CO_2_ reached its maximum in the nominal 2–3 nm pore models. These maxima may be influenced by model geometry and normalization because the probe-accessible pore volumes and surface areas after annealing were not quantified. In the smaller 1 and 2 nm pore models, CO_2_ exhibits a pronounced competitive adsorption advantage. As the pore size increases to 3–4 nm, the relative adsorption capacity of CH_4_ increases, thereby narrowing the adsorption difference between CH_4_ and CO_2_. These findings provide molecular-scale insights into pore-size-dependent equilibrium CH_4_/CO_2_ adsorption in experimentally informed organic-matter nanopores, offering a reference for future evaluations of CO_2_-enhanced gas recovery and storage in coal-measure shale.

## 1. Introduction

Coal-measure shale gas represents an important target for the synergistic development of deep coal-measure gas resources [1]. The Taiyuan and Shanxi formations in the Qinshui Basin contain well-developed marine–continental transitional coal-bearing deposits, in which dark mudstone and shale are interbedded with coal seams, sandstone, and limestone. This lithological assemblage provides a favorable geological basis for the joint exploration and co-production of coalbed methane and shale gas [2,3]. Compared with marine shale, coal-measure shale is generally dominated by type III kerogen, characterized by a low degree of aromatic condensation and strong structural heterogeneity [4,5,6]. Its nanopores not only serve as the principal adsorption and storage space for CH_4_ but also constitute critical interfaces for CO_2_-induced CH_4_ displacement [7,8,9]. Therefore, clarifying the relationships among the molecular structure of organic matter, pore-size scale, and competitive CH_4_/CO_2_ adsorption is fundamental to evaluating the potential of CO_2_-enhanced gas recovery (CO_2_-EGR) and CO_2_ sequestration in coal-measure shale [10].

Extensive studies have been conducted on coal-measure gas resource evaluation, pores associated with organic matter and clay minerals, molecular model construction, and CH_4_/CO_2_ adsorption behavior. These studies have demonstrated that pore size, pressure, temperature, and mineral composition exert significant effects on gas adsorption and transport [11,12,13,14]. Nevertheless, several limitations remain. Organic-matter pores smaller than 10 nm are difficult to characterize directly because of observational resolution constraints and therefore often require investigation using molecular models [15,16,17]. Previous GCMC investigations have demonstrated the importance of pore size and kerogen structure in gas adsorption, including CH_4_/CO_2_ competitive adsorption in systems containing micropores and mesopores [18,19,20]. However, less attention has been given to incorporating quantitative distributions of HRTEM-derived aromatic structural categories into the construction of pore-wall models for systematic pore-size comparisons. The present study therefore focuses on pore-size-dependent adsorption within an experimentally informed short-chain aromatic structural framework. Consequently, the pore-size-dependent effects of “short-chain aromatic” structures on competitive CH_4_/CO_2_ adsorption remain poorly understood [21,22,23,24].

To address these issues, coal-measure shale from the eastern Yushe Block of the Qinshui Basin was investigated in this study. Aromatic lattice fringes were extracted through HRTEM image processing, binarization, and vectorization, enabling the quantitative identification of 1 × 1, 2 × 2, and 3 × 3 short-chain aromatic units and their orientation characteristics. The experimentally determined structural proportions were subsequently incorporated into cylindrical organic-matter pore models with pore sizes of 1–4 nm. Following geometry optimization and annealing, grand canonical Monte Carlo (GCMC) simulations were performed to investigate the single-component adsorption of CH_4_ and CO_2_ and the competitive adsorption of an equimolar CH_4_/CO_2_ binary mixture at temperatures of 288–308 K and pressures of 0.25–8 MPa. The methodological contribution of this study is the use of HRTEM-derived aromatic structural categories and their relative abundances to inform the construction of organic-matter pore-wall models based on an experimentally characterized precursor molecular structure. The resulting simulations examine pore-size-dependent competitive adsorption within this specified structural framework, rather than independently isolating the effects of aromatic condensation or ordering.

## 2. Geological Setting

**Figure 1:**
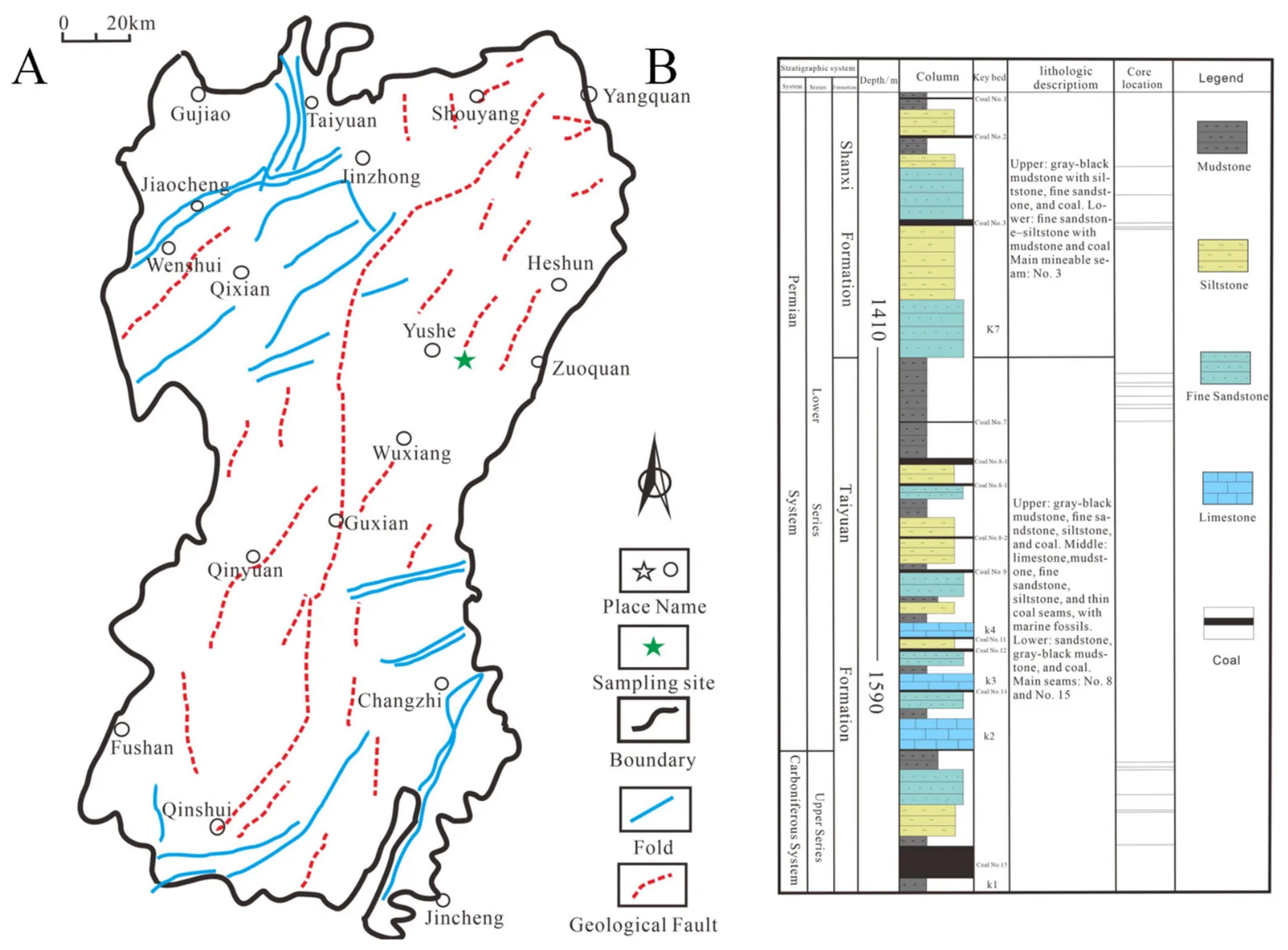
Tectonic framework map of the Qinshui Basin (**A**) and comprehensive stratigraphic column (**B**).

The Qinshui Basin is located in southeastern Shanxi Province and tectonically lies within the central belt of the North China Platform. It is an intermontane fault-depression basin formed by differential fault-block uplift following Late Paleozoic coal formation, and represents one of the most important coal-bearing basins and coal-measure gas exploration and development areas in China [25,26,27]. The eastern and western margins of the basin are controlled by the Taihang Uplift and the Lüliang Uplift, respectively. Overall, the basin is characterized by a relatively simple tectonic framework and favorable conditions for hydrocarbon preservation. The study area is situated in the eastern Yushe Block in the central-eastern Qinshui Basin, adjacent to the Yushe–Zuoquan deep coal-measure gas exploration belt. The coal-bearing strata are deeply buried, and coal seams are extensively interbedded with mudstone and shale (Figure 1), providing a favorable geological basis for the integrated evaluation of deep coalbed methane and coal-measure shale gas resources [28]. The Taiyuan Formation in the eastern Yushe–Zuoquan area is generally buried at depths of approximately 1100–1700 m. Regional geothermal investigations have also revealed spatially variable reservoir temperatures in the Qinshui Basin, including relatively low geothermal gradients in its eastern areas and an average No. 3 coal-reservoir temperature of approximately 30.6 °C.

The principal coal-bearing strata in the study area comprise the Upper Carboniferous Taiyuan Formation and the Lower Permian Shanxi Formation. Both formations were deposited above the Ordovician paleoweathering crust and together constitute a typical marine–continental transitional coal-bearing depositional system. The Taiyuan Formation was deposited in a composite setting comprising an epicontinental carbonate platform, barrier coast, and delta [29]. Its lithology consists predominantly of dark-gray to grayish-black mudstone and shale, sandy mudstone, siltstone, fine-grained sandstone, limestone, and coal seams. Influenced by repeated marine transgressions and regressions, the Taiyuan Formation exhibits pronounced depositional cyclicity and contains shallow-marine, tidal-flat, barrier-island, and lagoonal facies. In particular, tidal-flat–lagoonal environments favored the deposition of fine-grained argillaceous sediments and the preservation of organic matter [30,31]. The mudstone and shale intervals are generally 20–70 m thick, with greater thicknesses in the Yushe–Zuoquan area, where they are closely associated with coal seams.

The Shanxi Formation conformably overlies the Taiyuan Formation and was deposited in a fluvial-dominated deltaic and lower delta-plain system. It is composed mainly of grayish-black mudstone, sandy mudstone, siltstone, fine-grained sandstone, and coal seams, and contains distributary-channel, interdistributary-bay, and peat-swamp microfacies. The relatively low-energy hydrodynamic conditions in the interdistributary bays and peat swamps favored the accumulation of fine-grained sediments and terrestrial organic matter, making these environments the principal depositional settings for dark mudstone, shale, and coal seams in the Shanxi Formation. Coal seams Nos. 3, 8-1, and 15, together with the mudstone and shale in their roofs and floors, are relatively stable throughout the study area. The frequent interbedding of coal seams with mudstone and shale imparts typical marine–continental transitional characteristics to the sources of organic matter, pore structures, and gas adsorption behavior of the coal-measure shale [32,33,34,35,36]. Therefore, the study area is representative for investigating the organic-matter structure of deep coal-measure shale and the adsorption mechanisms of CH_4_ and CO_2_.

## 3. Materials and Methods

### 3.1. Sample Collection

The study area is located in the central-eastern part of the Qinshui Basin, within the Yushe–Zuoquan area (Figure 1). This area forms part of a nationally designated coalbed methane development zone and is a key target for coalbed methane and shale gas exploration. Compared with the southern Qinshui Basin, the central part of the basin generally has burial depths exceeding 1000 m and is therefore an important region for the exploration and development of deep coalbed methane. Drilling and logging data indicate that mudstone and shale are well developed in this area and exhibit favorable gas-logging responses. Based on drilling, mud-logging, and well-logging data, a total of 19 core samples were collected. These comprised five mudstone–shale samples from the roof and floor strata of the No. 3 coal seam in the Shanxi Formation, seven shale samples from the transitional interval between the Shanxi and Taiyuan formations, and seven samples from the Taiyuan Formation. The locations of the wells and sampling intervals are shown in Figure 1.

### 3.2. Experimental Methods

A total of 19 samples were collected in this study. Immediately after collection, the samples were sealed and transported to the laboratory. All samples were washed with distilled water, freeze-dried, and pulverized prior to subsequent analyses. A series of fundamental physicochemical characterization experiments were then conducted. For the preliminary screening and characterization of organic-rich samples, total organic carbon (TOC) contents were determined using an infrared carbon–sulfur analyzer in accordance with the Chinese national standard GB/T 19145–2003, Determination of Total Organic Carbon in Sedimentary Rocks. Mineralogical compositions were analyzed using a Rigaku Ultima IV powder X-ray diffractometer (Rigaku Corporation, Tokyo, Japan) in accordance with the Chinese petroleum and natural gas industry standard SY/T 5163–2010. Fourier-transform infrared spectroscopy (FTIR) was performed using a Bio-Rad FTS 165 spectrometer (Bio-Rad Laboratories, Inc., Hercules, CA, USA). Mineralogical and pore-structure characteristics were examined by field-emission scanning electron microscopy (FE-SEM) using Tescan MIRA 3 (TESCAN GROUP, a.s., Brno, Czech Republic) and JEOL JSM-7610F (JEOL Ltd., Tokyo, Japan) instruments. Organic elemental compositions were determined using an Elementar elemental analyzer (Elementar Analysensysteme GmbH, Langenselbold, Germany). Method for the Separation of Kerogen from Sedimentary Rocks. Organic elemental compositions were determined using an Elementar elemental analyzer. X-ray photoelectron spectroscopy (XPS) measurements of the organic-matter samples were conducted using a Thermo Fisher Scientific (Waltham, MA, USA) ESCALAB 250Xi spectrometer.

In addition, ^13^C nuclear magnetic resonance (^13^C NMR) spectra were acquired using a Bruker 400 MHz superconducting NMR spectrometer (Bruker BioSpin GmbH & Co. KG, Ettlingen, Germany) [37]. Among the investigated samples, QS-03 (TOC = 11.20%) was selected for organic-matter extraction, and the resulting demineralized specimen QS-03D was used for HRTEM analysis. Because the ^13^C NMR spectrum of QS-03D exhibited insufficient reproducibility, the previously characterized C_177_H_160_N_2_O_8_S molecular structure derived from Xishan Coalfield organic matter [38] was adopted as the precursor framework. FTIR and XPS observations of QS-03D and the HRTEM-derived aromatic-fringe distribution provided additional structural context for the pore-model construction.

High-resolution transmission electron microscopy constituted one of the key experimental techniques employed in this study. Because the spatial resolution of FIB-SEM imaging is limited to approximately 19.5 nm, pores smaller than 20 nm cannot be effectively resolved using this method. Therefore, HRTEM observations were performed to characterize smaller organic-matter pores and the molecular-scale structure of the organic matter. The experiments were conducted using a JEOL JEM-200F transmission electron microscope (JEOL Ltd., Tokyo, Japan). HRTEM generates microscopic images through the interaction of a high-energy electron beam with the specimen. Owing to the short wavelength of electrons, this technique provides substantially higher spatial resolution and enables direct observation of both surface and internal structural features. Transmission electron microscopy can be used to characterize material structures and surface morphologies at the nanometer scale and provides high-resolution information on lattice structures, atomic arrangements, and chemical compositions. In the analysis of coal and organic-matter microstructures, HRTEM images allow lattice fringes to be directly observed at or near the atomic scale. Lattice fringes of different dimensions correspond to different aromatic structural units; therefore, their dimensional and morphological characteristics can be used to characterize the distribution of aromatic clusters in coal and organic matter. The size ranges corresponding to the different aromatic structural units are summarized in Table 1.

During HRTEM specimen preparation, the powdered shale organic matter was distributed nonuniformly on the copper grid, with a relatively thick central region and thinner peripheral regions. Consequently, some lattice-fringe images acquired from the thicker regions exhibited noticeable distortion. Therefore, lattice-fringe images obtained from the edges of the specimens were preferentially selected for analysis, as these regions provided clearer and more uniform lattice information.

### 3.3. Construction of HRTEM-Constrained Organic-Matter Pore Models and GCMC Adsorption Simulation Settings

Previous low-temperature N_2_ adsorption and field-emission scanning electron microscopy studies have shown that nanopores in shale are generally smaller than 10 nm and are predominantly distributed within the range of 2–4 nm. Morphologically, these pores commonly occur as circular or elliptical voids [38]. Free CH_4_ molecules can be stored in relatively large pores, whereas adsorbed CH_4_ is mainly accommodated within circular or elliptical organic-matter pores. To investigate CH_4_ adsorption in nanoscale organic-matter pores, organic-matter fragments were used to construct pore models with diameters of 1–4 nm based on the lattice-fringe length distribution and the two-dimensional molecular structure of the organic matter. These models were subsequently employed for molecular simulations of CH_4_ adsorption [39,40].

**Figure 2:**
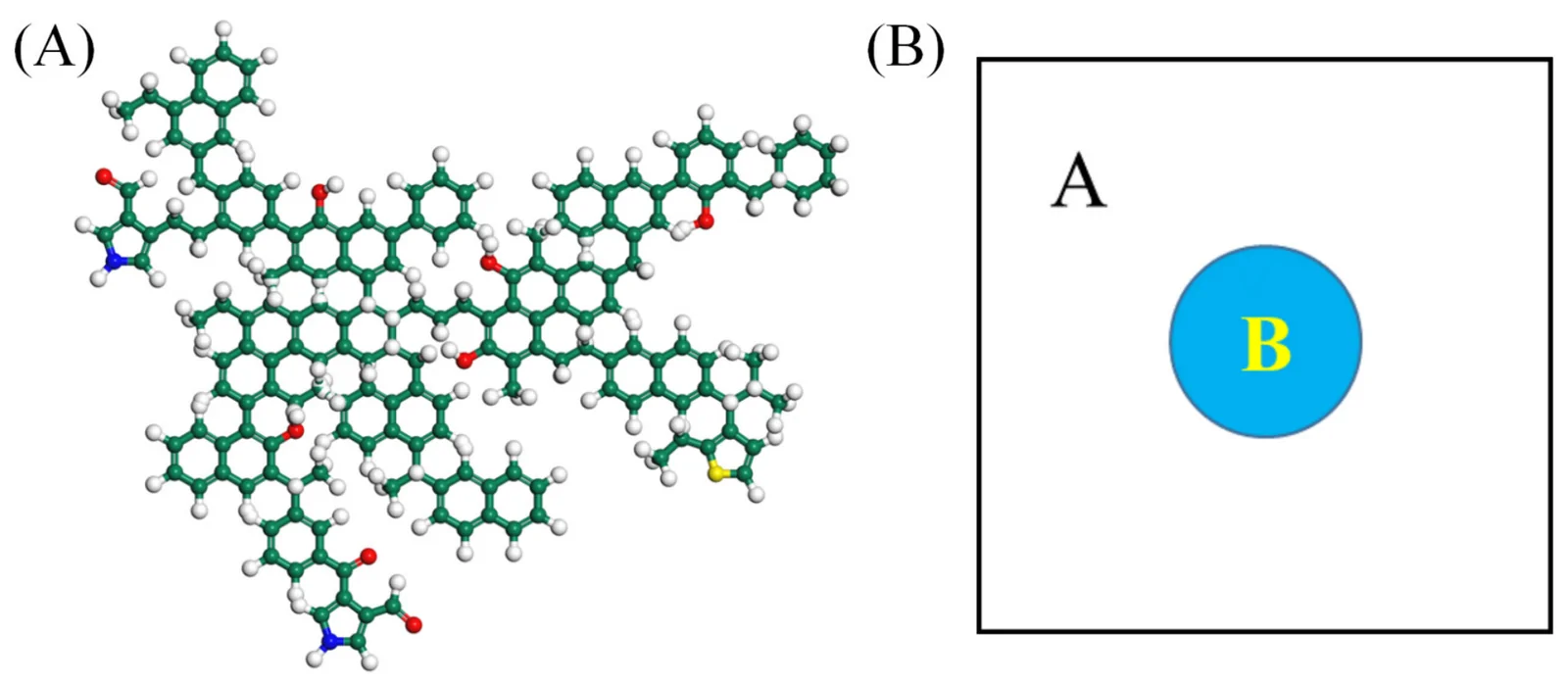
Construction of the organic-matter pore models. (**A**) Two-dimensional organic-matter molecular structure with the molecular formula C_177_H_160_N_2_O_8_S. (**B**) Schematic illustration of pore-model construction, where the white region labeled A represents the organic-matter-filled region, and the blue circular region labeled B represents the cylindrical pore space.

The two-dimensional organic-matter molecular structure used in this study is shown in Figure 2A, and its molecular formula is C_177_H_160_N_2_O_8_S. This molecular structure served as the basic structural unit for constructing the organic-matter pore models with different pore diameters. HRTEM lattice-fringe analysis showed that the number ratio of 1 × 1, 2 × 2, and 3 × 3 aromatic units in the organic matter was approximately 8:4:3. Accordingly, the two-dimensional organic-matter model was divided into a series of molecular fragments while maintaining this proportional relationship. The aromatic-unit proportions derived from the lattice-fringe length analysis were used as quantitative constraints for the pore-wall composition. The experimentally measured orientation distributions were used to characterize the structural ordering of the organic matter and were not imposed as angular constraints during pore-model construction. The pore-model construction procedure was as follows. The previously established precursor molecular structure derived from Xishan Coalfield organic matter [38] was constrained using elemental analysis, XPS, and ^13^C NMR data obtained from that reference material, rather than from QS-03D. Its aromatic carbon fraction (75.71%) and aromatic bridgehead-to-peripheral carbon ratio (0.196) were close to the corresponding experimental values for the Xishan reference material (75.57% and 0.195, respectively). The calculated O/C and N/C ratios were also close to the measured values, although discrepancies remained in H/C and S/C. These comparisons primarily validate selected characteristics of the precursor molecular model; independent quantitative verification of the functional-group distributions in the annealed 1–4 nm pore structures was not performed.

A cylindrical region with a diameter of c and a length of b was inserted into a rectangular simulation cell with a side length of a and a thickness of b. The space between the cylindrical region B and the surrounding simulation cell, denoted as region A, was filled with organic-matter molecular fragments. Cylindrical pore models with different diameters were constructed using this procedure, and the packing density of the organic matter in region A was set to 1.40 g cm^−3^. A schematic illustration of the model-construction procedure is presented in Figure 2B.

The model-filling calculation was performed using the following equation:

(1) $$\rho_{A}=\frac{\sum_{i=1}^{n}N_{i}M_{i}}{N_{\mathrm{Av}}V_{A}}$$

where ρA is the packing density of the organic-matter-filled region A (g/cm^3^); N_i_ is the number of atoms of element i in region A; M_i_ is the molar mass of element i (g/mol); n = 5 represents the five elements C, H, N, O, and S; N_Av_ is Avogadro’s constant (6.02214076 × 10^23^ mol^−1^); and V_A_ is the volume of the organic-matter-filled region A (cm^3^).

Taking the 1 nm pore model as an example, a rectangular simulation cell with a side length of 5 nm and a thickness of 1.5 nm was constructed, into which a carbon nanotube with a diameter of 1 nm was inserted. Shale organic-matter fragments were then filled into the space between the carbon nanotube and the rectangular cell [41,42]. After subdivision of the aromatic-ring structures in the shale organic matter, the number ratio of the 1 × 1, 2 × 2, and 3 × 3 aromatic units in the 1 nm pore model was approximately 8:4:3, consistent with the HRTEM-derived statistical distribution. A total of 12 two-dimensional organic-matter molecular fragments were used to complete the filling, and the density of the filled organic-matter region was set to 1.40 g/cm^3^.

**Figure 3:**
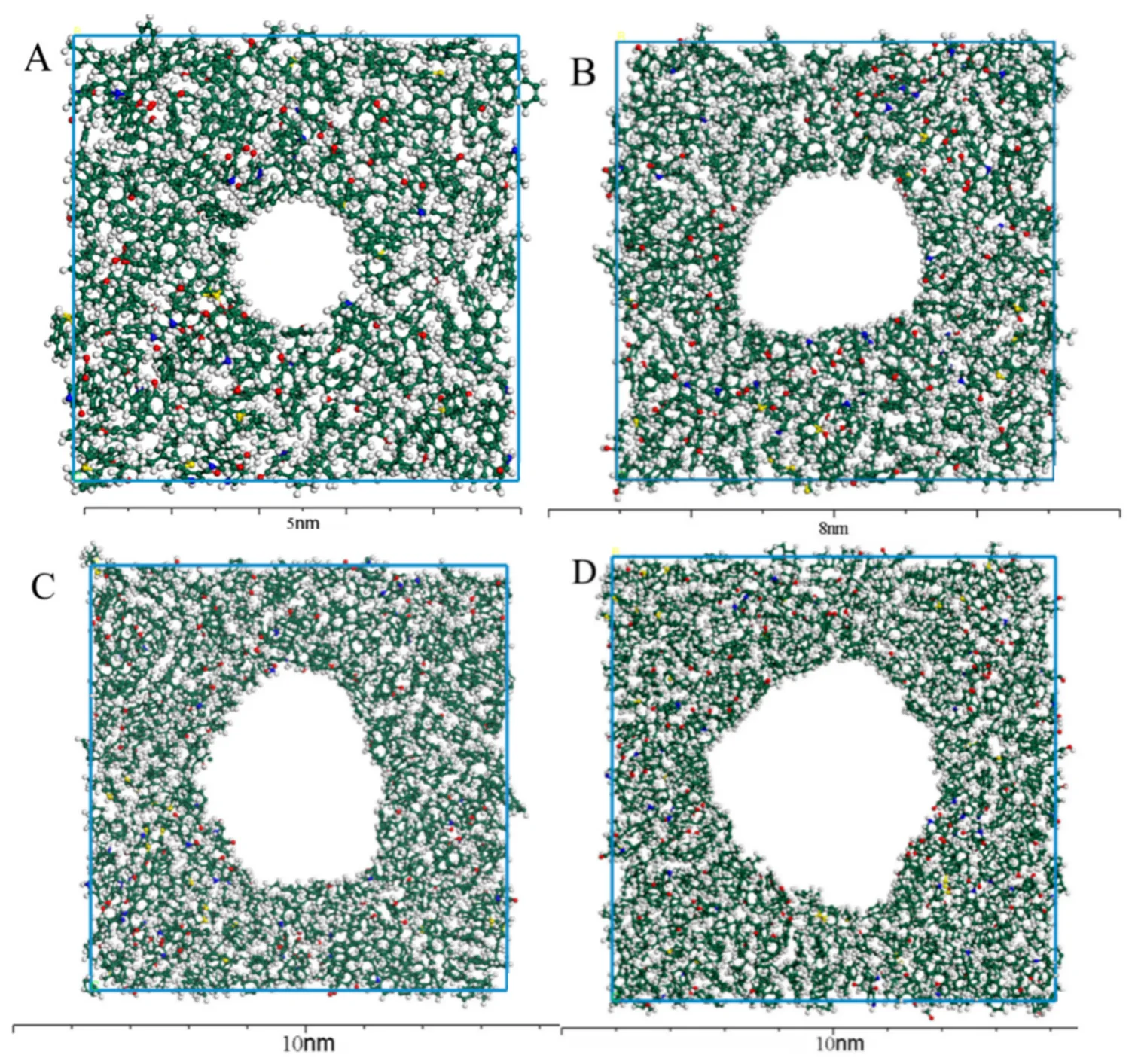
Depictions of (**A**) 1 nm cylindrical hole, (**B**) 2 nm cylindrical hole, (**C**) 3 nm cylindrical hole, and (**D**) 4 nm cylindrical hole.

Using the same procedure, cylindrical pore models with pore sizes of 1, 2, 3, and 4 nm were constructed. After model construction, geometry optimization and annealing treatments were performed to improve structural stability [43,44]. The resulting models are shown in Figure 3.

For the 2 nm cylindrical pore model, a square simulation cell with a side length of 6 nm and a thickness of 1.5 nm was constructed and filled with 15 two-dimensional organic-matter molecular fragments. For the 3 nm cylindrical pore model, the simulation cell had a side length of 7 nm and a thickness of 1.5 nm, and contained 21 two-dimensional organic-matter molecular fragments. For the 4 nm cylindrical pore model, a simulation cell with a side length of 8 nm and a thickness of 1.5 nm was constructed and filled with 26 two-dimensional organic-matter molecular fragments. The density of the organic-matter-filled region was maintained at 1.40 g/cm^3^ for all three models. For pore-size classification, the nominal 1 nm pore was regarded as a micropore (d < 2 nm), whereas the nominal 2, 3, and 4 nm pores were classified as mesopores (2 nm ≤ d < 50 nm). Based on the initial cylindrical geometry, the nominal pore volumes of the 1, 2, 3, and 4 nm models were 1.178, 4.712, 10.603, and 18.850 nm^3^, respectively. The corresponding nominal internal pore-wall areas were 4.712, 9.425, 14.137, and 18.850 nm^2^, with geometric porosities of 3.14%, 8.73%, 14.43%, and 19.63%. The organic-matter masses estimated from the target packing density and nominal filled-region volumes were 5.09 × 10^−20^, 6.90 × 10^−20^, 8.81 × 10^−20^, and 1.08 × 10^−19^ g, respectively. These values characterize the initial model geometry rather than the probe-accessible properties of the annealed pore structures.

The HRTEM-derived aromatic-unit distribution was used to guide the selection of molecular fragments, while the elemental composition and representative functional groups were based on the experimentally characterized precursor molecular structure. The assembled and annealed pore models therefore represent statistically informed three-dimensional structures rather than direct reconstructions of the two-dimensional HRTEM observations.

Following model construction, geometry optimization was performed using the Geometry Optimization function in the Forcite module of Materials Studio. The Smart algorithm was employed with the convergence quality set to Fine and the maximum number of iterations set to 1000. The DREIDING force field was adopted [44]. Both electrostatic and van der Waals interactions were calculated using the atom-based summation method, with a cutoff distance of 12.5 Å. The 12.5 Å cutoff was applied during the Forcite-based structural optimization. The subsequent GCMC adsorption simulations were performed separately using the Sorption module. The 1.5 nm cell dimension represents the axial periodic length of the cylindrical pore models and should be distinguished from the interaction cutoff used during structural optimization. This molecular-mechanics optimization enabled the models to reach local minima on the potential-energy surface.

To further approach the global minimum-energy state over the entire potential-energy surface, annealing molecular dynamics optimization was subsequently conducted using the Anneal function in the Forcite module. The simulations were performed in the NVT ensemble using the DREIDING force field. Electrostatic and van der Waals interactions were both evaluated using the atom-based summation method. The temperature was increased from an initial value of 300 K to 600 K in increments of 60 K and was then decreased to 300 K at the same rate. The total simulation time was 200 ps, with the default time step of 1 fs. Temperature was controlled using the Nosé thermostat, and 10 annealing cycles were performed.

At the end of each annealing cycle, the resulting configuration was subjected to molecular-mechanics optimization to ensure that it had reached a low-energy state. According to the minimum-energy criterion, the energy evolution curves were examined, and the annealed configuration with the lowest energy was selected as the optimal geometrical structure. During geometry optimization and annealing, the cylindrical pores with diameters of 2–4 nm underwent slight deformation, resulting in elliptical or irregularly rounded pore geometries [45,46]. For each nominal pore diameter, the selected low-energy annealed configuration was used as the representative structure for the subsequent adsorption calculations. The adsorption isotherms report the GCMC-derived adsorption quantities at the specified temperature and pressure conditions for these representative pore models, rather than averages over independently generated pore structures.

Compared with inorganic mineral components, shale organic matter generally exhibits a stronger gas adsorption capacity. The adsorption performance of shale organic matter is governed by its chemical composition and molecular structure, as well as by the physicochemical properties of the adsorbate gas. Organic-matter pores are widely regarded as the principal sites for gas adsorption and storage in shale. In general, a more highly developed pore system and smaller pore sizes result in a larger specific surface area, thereby enhancing the gas adsorption capacity of shale organic matter.

In this study, molecular simulations were performed to investigate the single-component adsorption characteristics of CH_4_ and CO_2_ in organic-matter pores under different temperature and pressure conditions, thereby elucidating the effects of temperature and pressure on the gas adsorption capacity of organic matter. The competitive adsorption behavior of a binary CH_4_/CO_2_ mixture was also simulated. The competitive adsorption behavior of the binary CH_4_/CO_2_ mixture was evaluated based on the component adsorption capacities and the corresponding CO_2_/CH_4_ adsorption selectivity. The adsorption of CH_4_ and CO_2_ in organic-matter nanopores was simulated using the Sorption module of Materials Studio under periodic boundary conditions [47,48]. The CH_4_ and CO_2_ molecules were treated as rigid molecular models, and the organic-matter pore models were also assumed to be rigid. During the simulations, only nonbonded interactions between the adsorbate molecules and the organic-matter pore walls were considered, including electrostatic and van der Waals interactions.

The adsorption simulations were performed using the optimized pore configurations obtained after geometry optimization and annealing. Although these preprocessing procedures allowed structural relaxation before adsorption, the pore-wall atoms remained fixed during the subsequent GCMC calculations. Therefore, pressure-dependent organic-matter swelling, adsorption-induced matrix deformation, and changes in pore accessibility were not explicitly considered. The calculated adsorption capacities and competitive adsorption behavior consequently represent the equilibrium adsorption characteristics of the pre-optimized, fixed pore structures rather than those of dynamically deformable organic-matter frameworks.

Single-component and binary competitive adsorption simulations of CH_4_ and CO_2_ were conducted at temperatures of 288, 298, and 308 K over a pressure range of 0.25–8 MPa. For the binary CH_4_/CO_2_ adsorption simulations, the bulk-gas composition was specified as an equimolar mixture, with yCH_4_ = yCO_2_ = 0.5. The nominal partial pressure of each component was defined as p_i_ = y_i_P, where P denotes the total pressure. The same gas-composition and pressure settings were applied to all pore models to ensure consistent comparisons of competitive adsorption behavior. The CO_2_/CH_4_ adsorption selectivity was defined as S_(CO2/CH4)_ = (xCO_2_/xCH_4_)/(yCO_2_/yCH_4_), where x_i_ and y_i_ represent the mole fractions of component i in the adsorbed and bulk-gas phases, respectively. The adsorbed-phase mole fractions were determined from the component adsorption amounts obtained in the binary simulations. For the equimolar mixture considered here, the selectivity simplifies to S_(CO2/CH4)_ = NCO_2_/NCH_4_, where N_i_ is the mean adsorbed number of component i under the same pore-size, temperature, and pressure conditions [49,50,51].

The adsorption volume (mL/cm^3^) was expressed as the equivalent gas volume corresponding to the GCMC-averaged adsorbed-molecule count per unit nominal cylindrical pore volume. The reference pore volume was defined as V_p_ = πd^2^L/4, where d is the nominal pore diameter and L is the pore length. The adsorption loading was treated as absolute adsorption without subtracting a bulk-gas contribution, and the same nominal-pore-volume normalization was used for all four models.

Detailed computational settings for the molecular modeling and GCMC adsorption simulations are provided in Sections S1–S5 of the Supplementary Materials. These include the preparation and geometry optimization of CH_4_ and CO_2_ molecules, construction and annealing of the organic-matter pore frameworks, nonbonded interaction settings, Monte Carlo sampling procedures, gas-mixture composition, and adsorption data processing. The binary CH_4_/CO_2_ simulations employed an equimolar gas mixture with a fixed molar ratio of 1:1 under all investigated pore-size, temperature, and pressure conditions.

## 4. Results and Discussion

### 4.1. HRTEM-Constrained Organic-Matter Pore Models with Short-Chain Aromatic Structures

The original HRTEM images of the organic-matter lattice fringes were affected by substantial noise, a narrow grayscale distribution, and poor fringe contrast, making direct identification and quantitative analysis difficult. Therefore, image processing was performed before lattice-fringe extraction. The main processing workflow comprised selection of the region of interest, fast Fourier transform (FFT), annular filtering, and inverse fast Fourier transform (IFFT). The processed images were required to exhibit a uniform background and clearly distinguishable lattice fringes.

**Figure 4:**
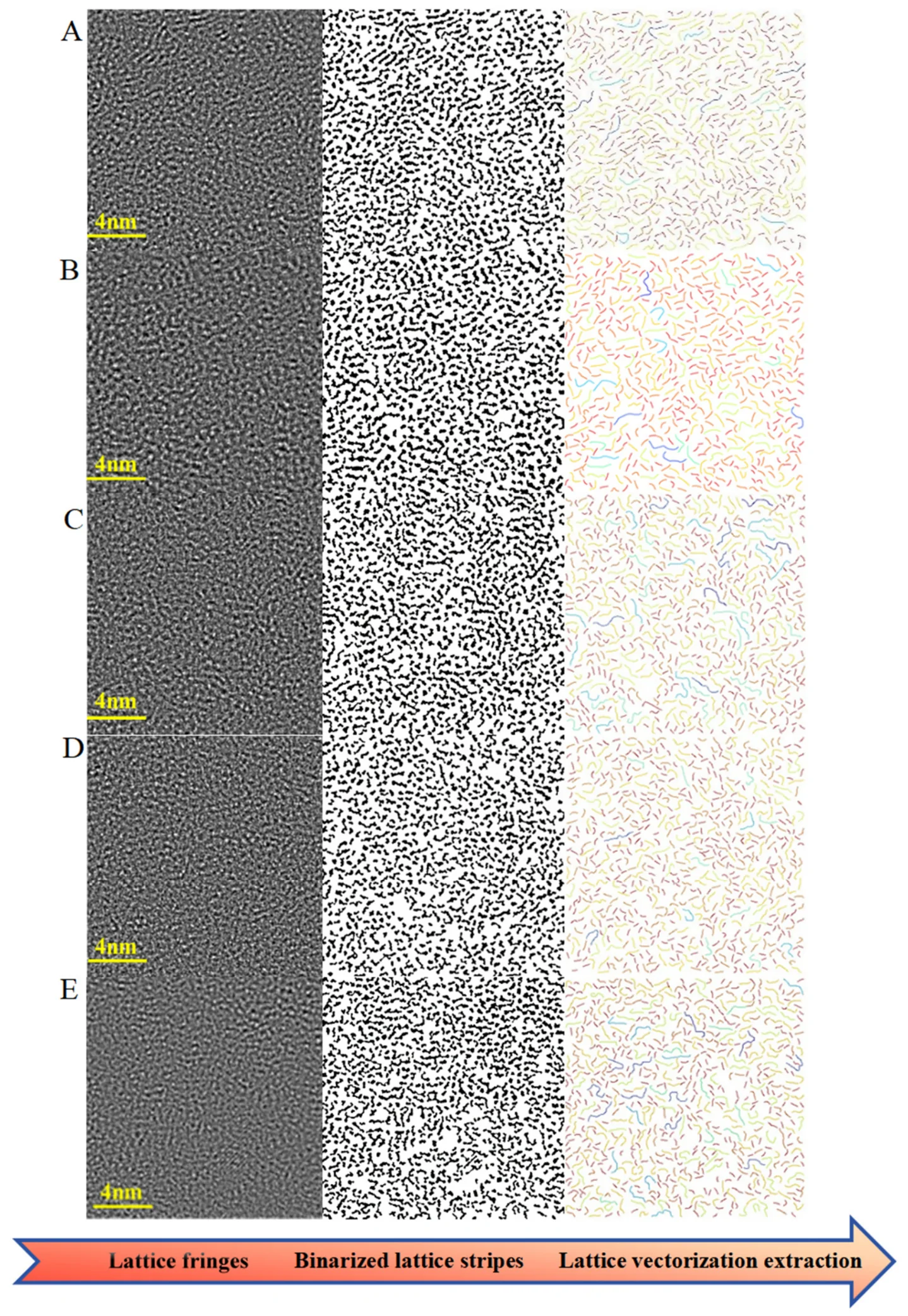
HRTEM lattice-fringe image processing results. (**A**–**E**) Five representative HRTEM image regions. From left to right, each row shows the original HRTEM image, the binarized lattice-fringe image, and the corresponding vectorized lattice-fringe map.

The processed images were subsequently equalized and binarized using ImageJ (version 1.53, National Institutes of Health, Bethesda, MD, USA), to obtain black-and-white lattice-fringe images. To quantitatively analyze the skeletal characteristics of the lattice fringes, the fringe information was further vectorized. Five HRTEM images acquired from the edges of the specimen were selected and labeled A–E. The binarized HRTEM images were imported into ArcGIS (version 10.8, Esri, Redlands, CA, USA), where the lattice fringes were manually traced and extracted. This procedure yielded the principal skeletal information of the aromatic-layer lattice fringes in the organic matter. In total, 3297 lattice fringes were extracted, and the results are presented in Figure 4.

To quantitatively characterize the molecular structure and composition of the organic matter, the lengths of the lattice fringes were measured. Based on the measured fringe lengths and the corresponding size ranges listed in Table 1, the lattice fringes were classified into different aromatic structural categories (1 × 1 to 8 × 8). The numbers and proportions of each category in the five HRTEM images are presented in Table 2.

The between-image standard deviations of the 1 × 1, 2 × 2, and 3 × 3 fringe proportions were 4.32, 4.26, and 1.39 percentage points, respectively, reflecting variation among the five selected HRTEM fields. Potential uncertainties associated with manual tracing and preferential selection of clearer specimen-edge regions were not independently quantified. The assigned aromatic structural categories should therefore be regarded as approximate fringe-length classes rather than unique molecular identifications.

The statistical results show that the lattice-fringe distributions in the five images were generally consistent. Short fringes were the most abundant, followed by medium-length fringes, whereas long fringes were relatively scarce. Among the different structural categories, 1 × 1 fringes were dominant, accounting for 41.5–52.9% of the total, with an average proportion of 48.4%. Thus, nearly half of all identified fringes corresponded to the 1 × 1 category, indicating that single benzene-ring units were the most prevalent aromatic structures in the organic matter.

The 2 × 2 and 3 × 3 fringes were the next most abundant categories. The proportion of 2 × 2 fringes ranged from 21.3% to 30.6%, with an average of 24.2%, whereas that of 3 × 3 fringes ranged from 16.4% to 19.8%, with an average of 18.2%. These results indicate that naphthalene- and anthracene-like aromatic units were also relatively well developed. Overall, lattice fringes with lengths of 0.30–1.14 nm accounted for more than 90% of the total. The 4 × 4 category was also present in the shale organic matter, with an average proportion exceeding 5%.

Although the vitrinite reflectance of the shale organic matter in the study area exceeded 1.8%, its degree of aromatic condensation remained relatively low, as reflected by the generally short lattice-fringe lengths. The number of fused aromatic rings was mainly concentrated between two and four, with shorter naphthalene-like fringes being dominant and anthracene-like fringes occurring secondarily. These aromatic structural interpretations were derived from the HRTEM lattice-fringe analysis of QS-03D and were not independently verified using its ^13^C NMR data because those measurements lacked sufficient reproducibility. To characterize the in-plane orientation of aromatic lattice fringes, the fringe orientations were treated as axial data, because directions separated by 180° represent the same unoriented structural axis. For each fringe consisting of n independently traced linear segments with orientation angles θ_i_, the doubled-angle trigonometric components were calculated using Equation (2).

(2) $$C_{2}=\sum_{i=1}^{n}\cos(2\theta_{i}),S_{2}=\sum_{i=1}^{n}\sin(2\theta_{i})$$

The mean axial orientation (LDM) was subsequently calculated using Equation (3).

(3) $$\mathrm{LDM}=[\frac{1}{2}a\tan2(S_{2},C_{2})]\mathrm{mod}\Pi$$

**Figure 5:**
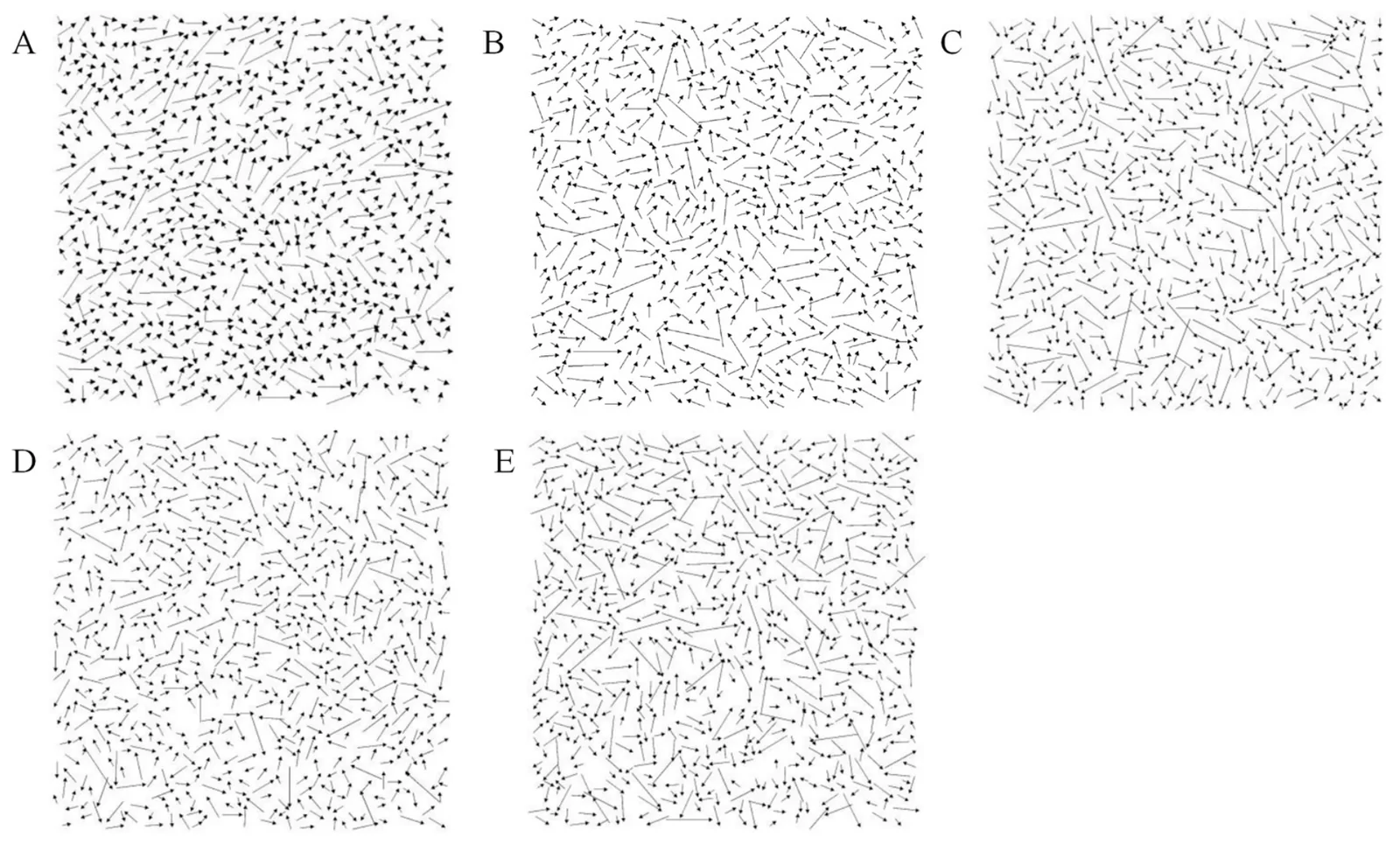
Schematic diagrams of aromatic lattice-fringe orientations in organic matter. (**A**–**E**) Lattice-fringe orientation maps corresponding to the five selected HRTEM image regions.

Here, *a*tan2 denotes the two-argument inverse tangent function, which correctly resolves the angular quadrant, while the modulo-π operation restricts the calculated axial orientation to the interval from 0° to 180°. All trigonometric calculations were performed in radians, and the resulting orientations were converted to degrees for graphical presentation. The corresponding lattice-fringe orientation diagrams are shown in Figure 5.

**Figure 6:**
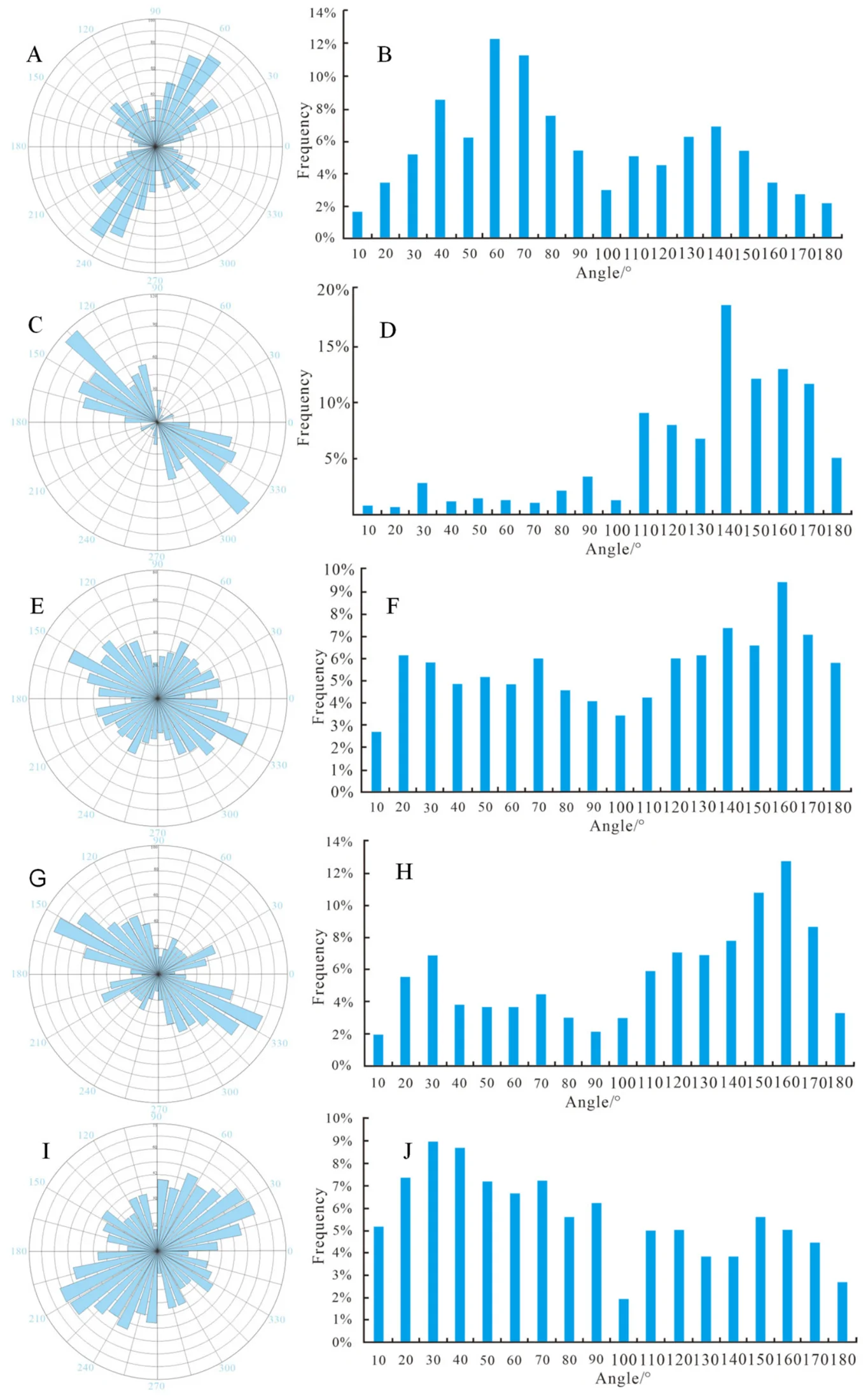
Orientation distributions of aromatic lattice fringes in five selected HRTEM image regions. (**A**,**B**), (**C**,**D**), (**E**,**F**), (**G**,**H**), and (**I**,**J**) correspond to the five image regions, respectively. The left panels show rose diagrams, and the right panels show the corresponding orientation-frequency histograms.

The axial orientation distributions of the organic-matter lattice fringes are presented in Figure 6. The histograms show orientation frequencies within 0–180°, whereas the rose diagrams display the corresponding axes in two opposite directions for visualization. Thus, angular sectors separated by 180° represent equivalent fringe orientations rather than independent preferred directions. The distributions vary among the five investigated image fields, indicating differences in local aromatic structural alignment.

In the first group, the lattice fringes exhibited an approximately “short-cross-shaped” distribution and were mainly concentrated within the angular ranges of 50–70° and 120–140°, indicating a certain degree of symmetry. The distributions within 280–330° and 105–150° were relatively weak, and only a small number of fringes occurred in the other directions. In the second group, the fringe orientations exhibited a pronounced unidirectional preference, with most fringes concentrated within 100–160° and the diagonally opposite range of 280–350°. This pattern indicates a strong directional alignment of the aromatic layers in this region.

In the third group, the lattice fringes were relatively uniformly distributed in all directions, although higher frequencies occurred within 150–160° and 330–340°. This result indicates that local preferred orientations remained present against an overall background of dispersed arrangement. In the fourth group, the fringes were predominantly concentrated within 130–160° and 320–340°, whereas their distributions in the other directions were more dispersed and occurred at relatively low frequencies. This pattern suggests a relatively well-defined dominant alignment direction in this region. In the fifth group, the lattice-fringe orientations exhibited the most uniform distribution, with fringes developed in nearly all directions. Nevertheless, relatively high frequencies were still observed within 20–40° and 200–220°.

Overall, the lattice fringes of the organic matter were not completely randomly distributed but exhibited varying degrees of preferred orientation in different regions. These results demonstrate that the aromatic structures of the coal-measure shale organic matter possess local structural ordering and pronounced spatial heterogeneity.

**Figure 7:**
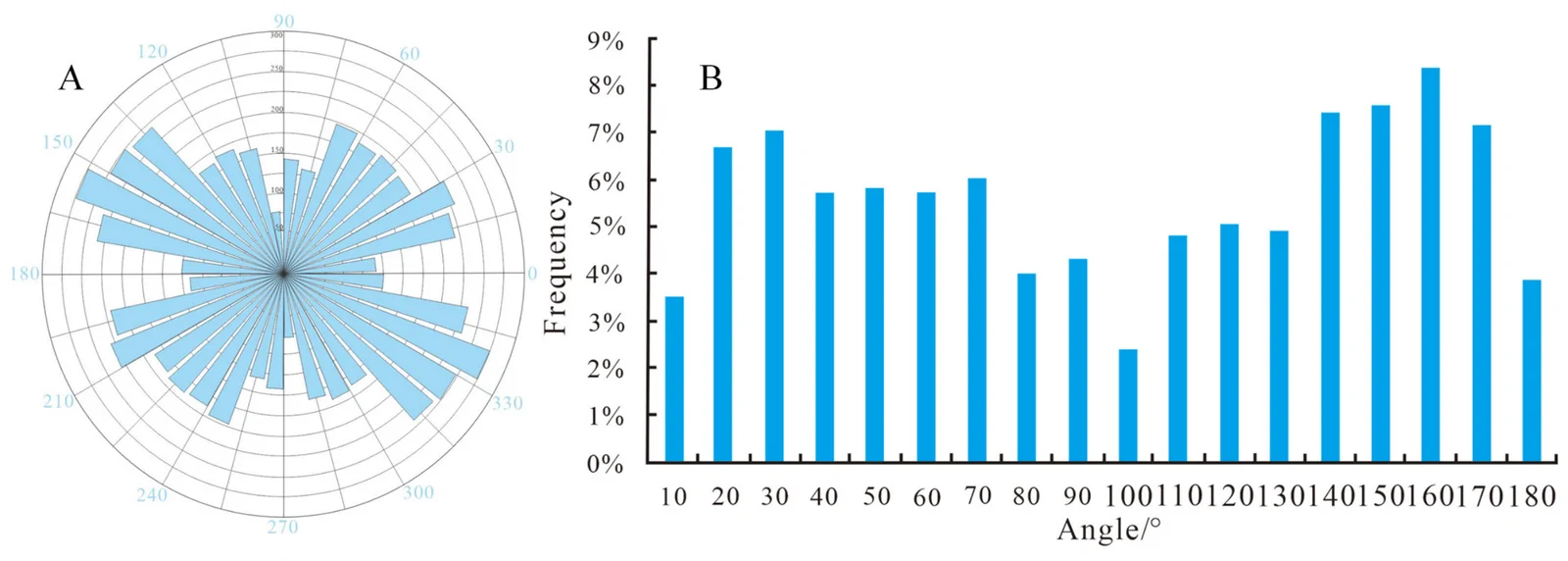
Overall orientation distribution of aromatic lattice fringes in organic matter. (**A**) Rose diagram of lattice-fringe orientations; (**B**) Corresponding orientation-frequency histogram.

As shown in Figure 7, the aromatic lattice fringes exhibited a broad axial orientation distribution across 0–180°, with a relatively high frequency in the 140–170° range and a secondary concentration around 20–30°. The most frequent orientation interval was 150–160°, accounting for approximately 8.3% of the fringes in the reported histogram. These results indicate local preferential alignment superimposed on a relatively dispersed aromatic structural arrangement.

These results indicate that the organic matter in the coal-measure shale is not dominated by highly condensed, large aromatic lamellae, but is primarily composed of short-chain aromatic units such as benzene, naphthalene, and anthracene. This structural characteristic gives rise to localized and heterogeneous adsorption sites on the organic-matter pore walls, thereby providing a molecular structural basis for the pore-size-dependent adsorption of CH_4_ and CO_2_ in 1–4 nm pores.

The orientation distributions in Figure 6 and Figure 7 primarily characterize the local ordering and structural heterogeneity of the experimental organic matter, whereas the quantitative HRTEM-derived information incorporated into the adsorption models was the relative abundance of the dominant aromatic structural units.

### 4.2. Pore-Size-Controlled Differences in Single-Component CH_4_ and CO_2_ Adsorption

**Figure 8:**
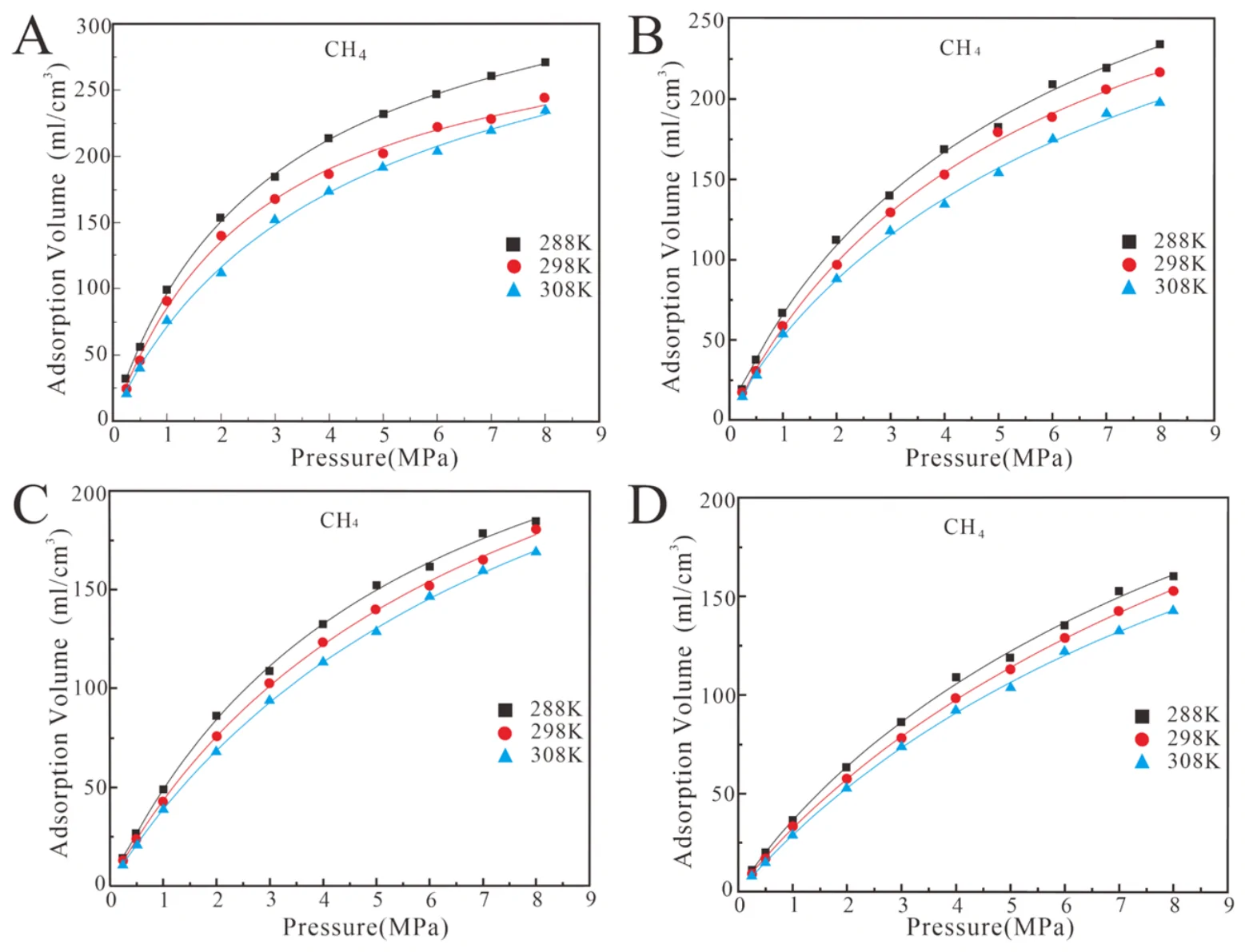
Isothermal curves of methane adsorption on organic matter pores of different sizes: (**A**) 1 nm; (**B**) 2 nm; (**C**) 3 nm; and (**D**) 4 nm.

**Figure 9:**
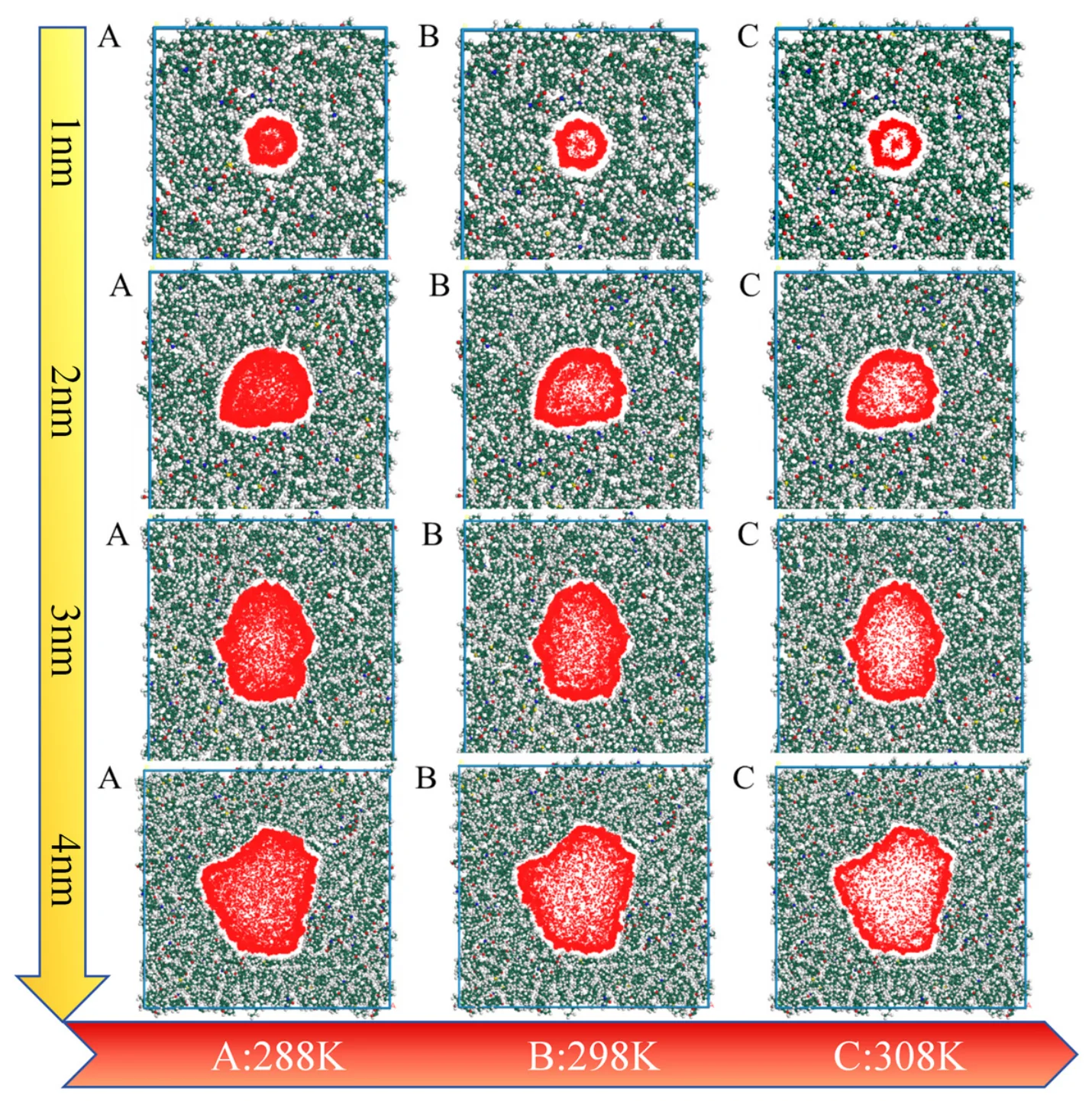
Spatial distributions of CH_4_ adsorption in organic-matter pores at 8 MPa. The four rows (top to bottom) represent pore diameters of 1, 2, 3, and 4 nm, respectively, while columns (**A**–**C**) correspond to 288, 298, and 308 K, respectively. Red spheres represent adsorbed CH_4_ molecules.

The CH_4_ adsorption simulation results are presented in Figure 8. Overall, the amount of CH_4_ adsorbed in the organic-matter pores increased progressively with increasing pressure, whereas it decreased with increasing temperature. As the pore size increased, the total adsorption capacity increased, while the adsorption capacity per unit pore volume decreased. This indicates that an increase in pore size enlarges the available pore volume and enhances the overall gas-storage capacity. However, the corresponding decrease in pore specific surface area weakens the adsorption capacity per unit pore volume. Notably, the volumetric adsorption capacity of the 1 nm pore was nearly twice that of the 4 nm pore. The spatial distribution maps of CH_4_ adsorption in the 1–4 nm pores are shown in Figure 9.

**Figure 10:**
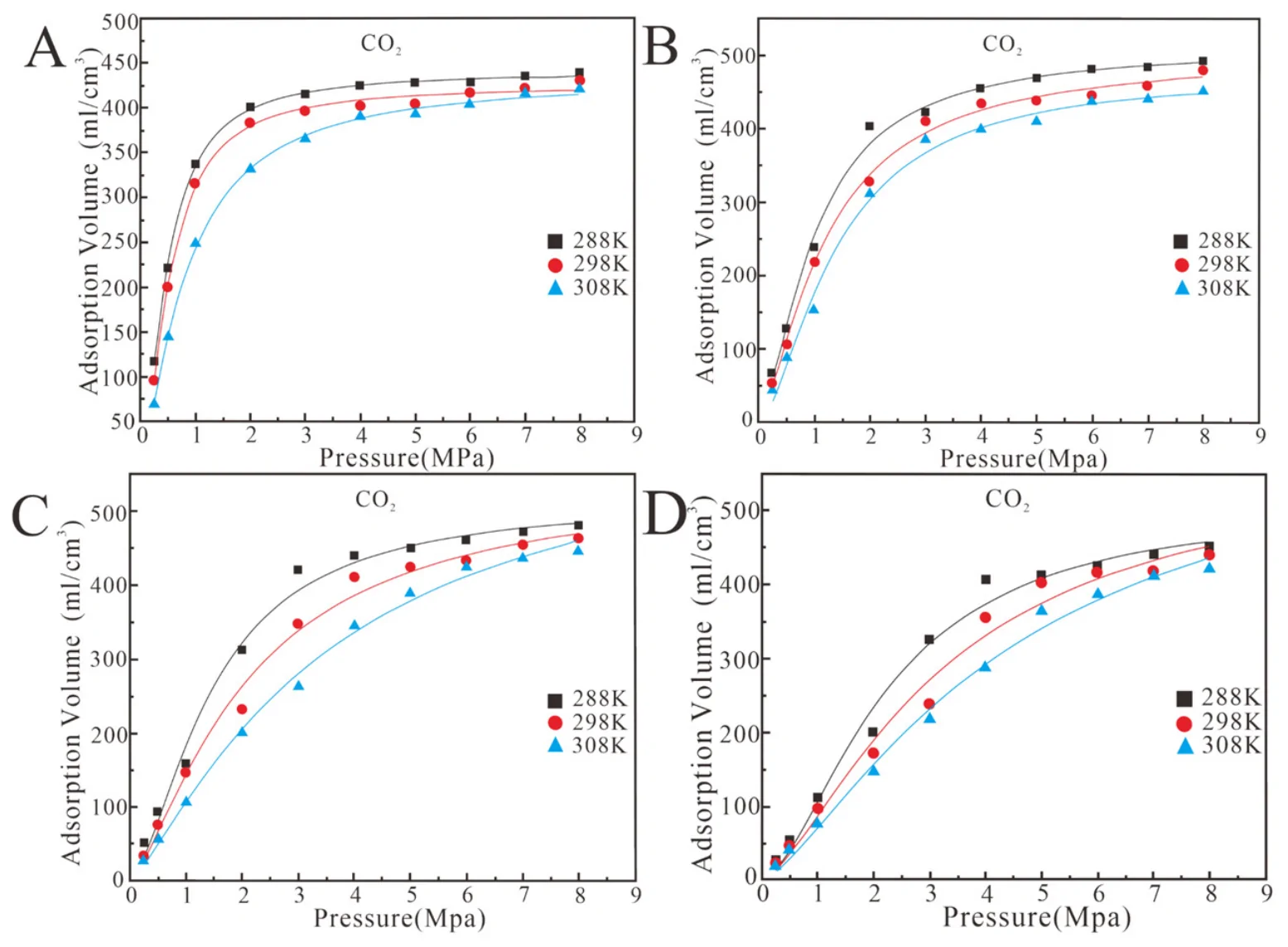
Isothermal curves of carbon dioxide adsorption on organic matter pores of different sizes: (**A**) 1 nm; (**B**) 2 nm; (**C**) 3 nm; and (**D**) 4 nm.

The single-component CO_2_ adsorption isotherms are presented in Figure 10. Similar to CH_4_ adsorption, the amount of CO_2_ adsorbed in the organic-matter pores increased with increasing pressure and decreased with increasing temperature. As the pore size increased, the pore volume and total CO_2_ adsorption capacity both increased. However, unlike CH_4_, whose volumetric adsorption capacity was highest in the 1 nm pore and decreased progressively with increasing pore size, the volumetric adsorption capacity of CO_2_ initially increased and then decreased. The highest CO_2_ adsorption capacity per unit pore volume was observed in the 2–3 nm pores.

**Figure 11:**
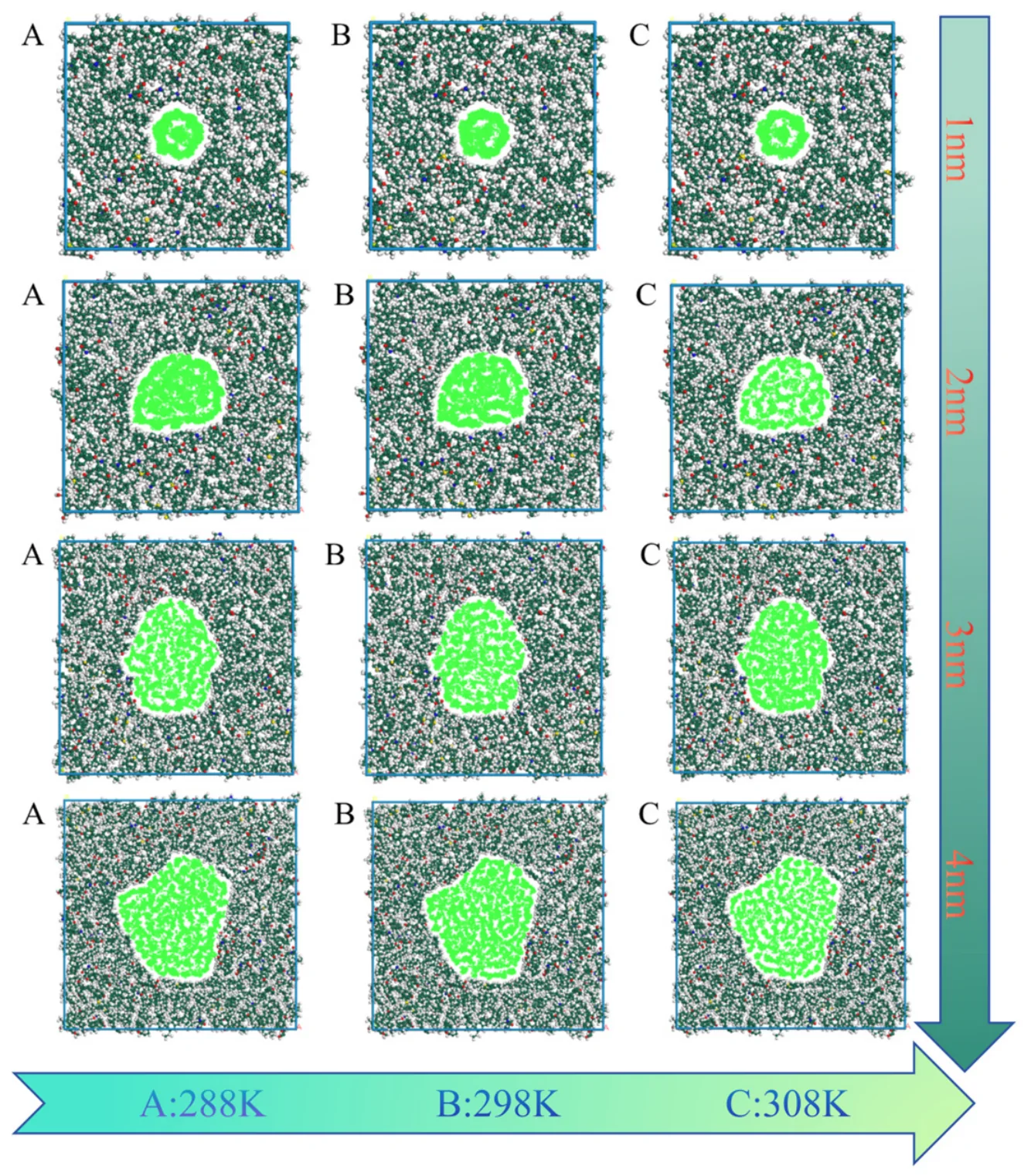
Spatial distributions of CO_2_ adsorption in organic-matter pores at 8 MPa. The four rows (top to bottom) represent pore diameters of 1, 2, 3, and 4 nm, respectively, while columns (**A**–**C**) correspond to 288, 298, and 308 K, respectively. Green spheres represent adsorbed CO_2_ molecules.

The relatively high CO_2_ volumetric adsorption capacity observed in the nominal 2–3 nm pores may reflect the combined effects of molecular confinement, available pore space, and interactions with the organic-matter pore walls. However, the probe-accessible pore volumes and surface areas of the annealed models were not independently quantified. Therefore, the observed adsorption maximum should be interpreted within the geometric and normalization conditions of the present model series. The spatial distribution maps of CO_2_ adsorption in the 1–4 nm pores are shown in Figure 11.

These results demonstrate that CH_4_ and CO_2_ respond differently to variations in pore size [51]. CH_4_ adsorption is governed predominantly by the micropore-filling mechanism, whereas CO_2_ adsorption is jointly controlled by the availability of adsorption sites on the pore walls and the accessible pore space.

### 4.3. Competitive Adsorption Characteristics and Interactions of Binary CH_4_/CO_2_ Mixtures

**Figure 12:**
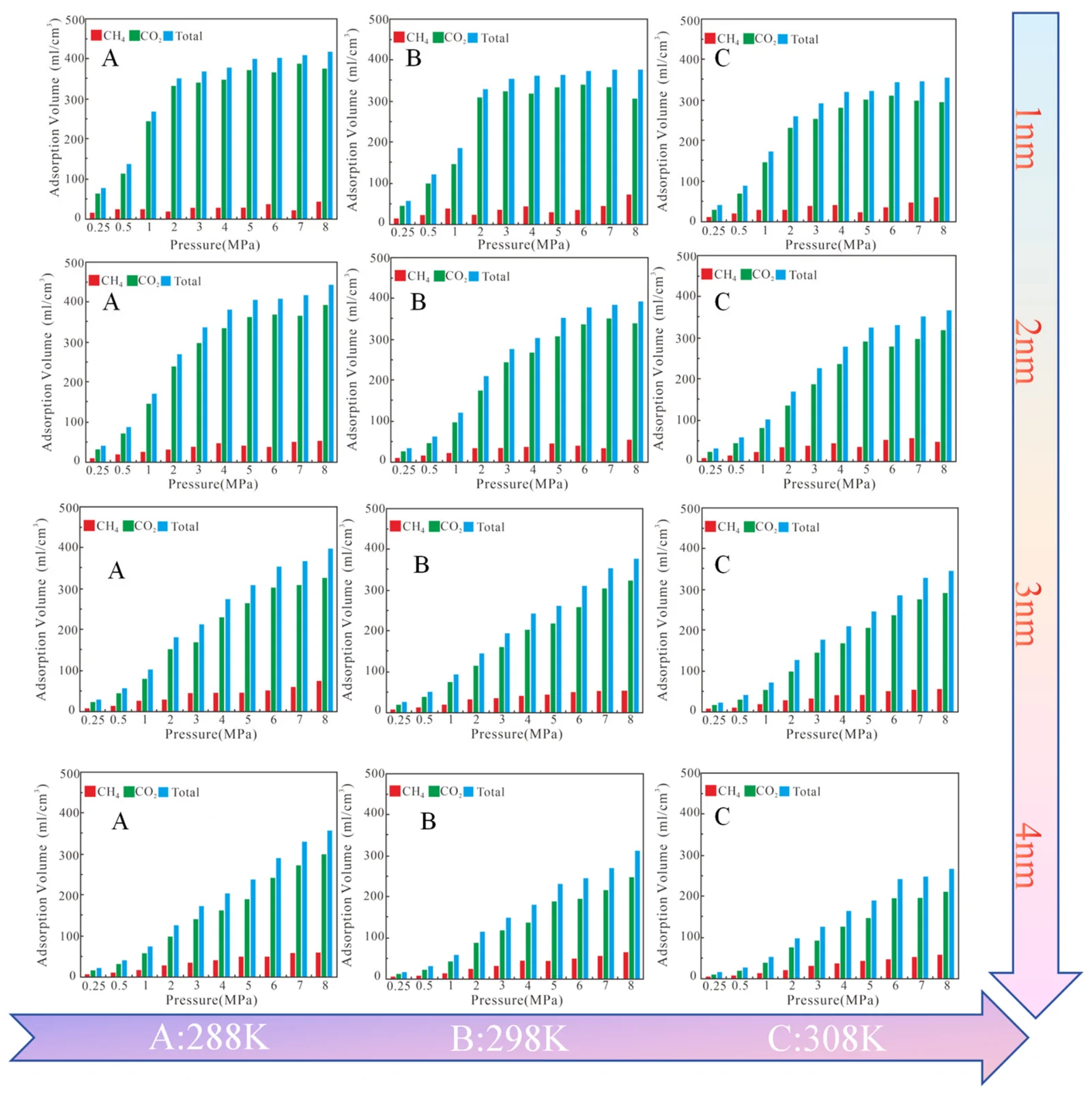
Comparison of CH_4_, CO_2_, and total adsorption volumes in binary competitive adsorption. The four rows (top to bottom) represent pore diameters of 1, 2, 3, and 4 nm, respectively, while columns (**A**–**C**) correspond to 288, 298, and 308 K, respectively. Red, green, and blue bars represent CH_4_, CO_2_, and total adsorption volumes, respectively, at different pressures.

Figure 12 presents the competitive adsorption results of the binary CH_4_/CO_2_ system as grouped bar charts, allowing direct comparison of the individual adsorption capacities of CH_4_ and CO_2_ and their combined adsorption capacity under different pore-size, temperature, and pressure conditions. Overall, the total adsorption capacity in the pores decreased with increasing temperature but increased with increasing pressure [52]. Similar to the adsorption behavior of CO_2_, the volumetric adsorption capacity of the binary system first increased and then decreased with increasing pore size, reaching a maximum in the 2 nm organic-matter pore and then decreasing as the pore size further increased.

When the pore size was 2 nm, the combined volumetric adsorption capacity of CH_4_ and CO_2_ was the highest, followed by those of the 1 and 3 nm pores, whereas the 4 nm pore exhibited the lowest combined adsorption capacity. In organic matter, a more developed pore system and smaller pore sizes generally correspond to a larger specific surface area and thus a stronger adsorption capacity. The combined volumetric adsorption capacity of CH_4_ and CO_2_ followed the order of 2 nm > 1 nm > 3 nm > 4 nm. This non-monotonic trend may reflect the combined effects of molecular confinement, accessible pore space, and pore-wall interactions. Although smaller pores generally provide stronger confinement, excessive spatial restriction in the 1 nm pore may reduce the space available for gas adsorption. However, because the probe-accessible pore volumes and surface areas of the optimized models were not independently quantified, the possible influence of pore-volume normalization cannot be excluded. Therefore, the higher volumetric adsorption capacity observed in the 2 nm pore may reflect the combined effects of molecular confinement, accessible pore space, and pore-wall interactions, rather than being solely determined by intrinsic adsorption affinity. Nevertheless, the potential influence of pore-volume normalization and molecular accessibility requires further quantitative evaluation.

At a given pore size, the adsorption capacities of both CH_4_ and CO_2_ were generally positively correlated with pressure and negatively correlated with temperature. An increase in system pressure increases the collision frequency between gas molecules and the pore surface, thereby enhancing the likelihood of van der Waals interactions and improving adsorption capacity. In contrast, an increase in temperature intensifies the thermal motion of gas molecules and increases their kinetic energy, making desorption from the pore surface more likely. The adsorption curves for the 1 and 2 nm pores show that the combined volumetric adsorption capacity of CH_4_ and CO_2_ increased rapidly at low pressure and gradually approached a plateau at higher pressure, indicating a saturation-like adsorption trend in these two smaller pore models. By contrast, the adsorption curves for the 3 and 4 nm pores display an approximately linear increase, suggesting that adsorption saturation had not yet been reached within the investigated pressure range.

At low pressure, only a small amount of CH_4_ was adsorbed. With increasing pressure, the increase in CH_4_ adsorption remained limited, whereas CO_2_ adsorption increased markedly. This indicates that the organic-matter pores exhibit strong adsorption selectivity toward the two gas species, and that adsorption of CO_2_ suppresses the adsorption of CH_4_ [49]. As shown in Figure 12, CO_2_ generally exhibited higher adsorption amounts than CH_4_ in the equimolar binary system. According to the selectivity definition, a higher CO_2_ adsorption amount corresponds to S(CO_2_/CH_4_) > 1, indicating preferential CO_2_ adsorption under the investigated conditions. The relative adsorption amounts of the two components varied with pore diameter, temperature, and pressure, reflecting corresponding changes in their competitive adsorption behavior. These observations are interpreted for the equimolar CH_4_/CO_2_ composition specified in the present simulations. Comparison with the single-component adsorption results further shows that the adsorption capacities of both CH_4_ and CO_2_ in the mixed system were lower than those in the corresponding single-component systems, confirming the occurrence of competitive adsorption. Moreover, the reduction in CH_4_ adsorption was much greater than that in CO_2_ adsorption, indicating that the competitive adsorption ability of CO_2_ is significantly stronger than that of CH_4_.

**Figure 13:**
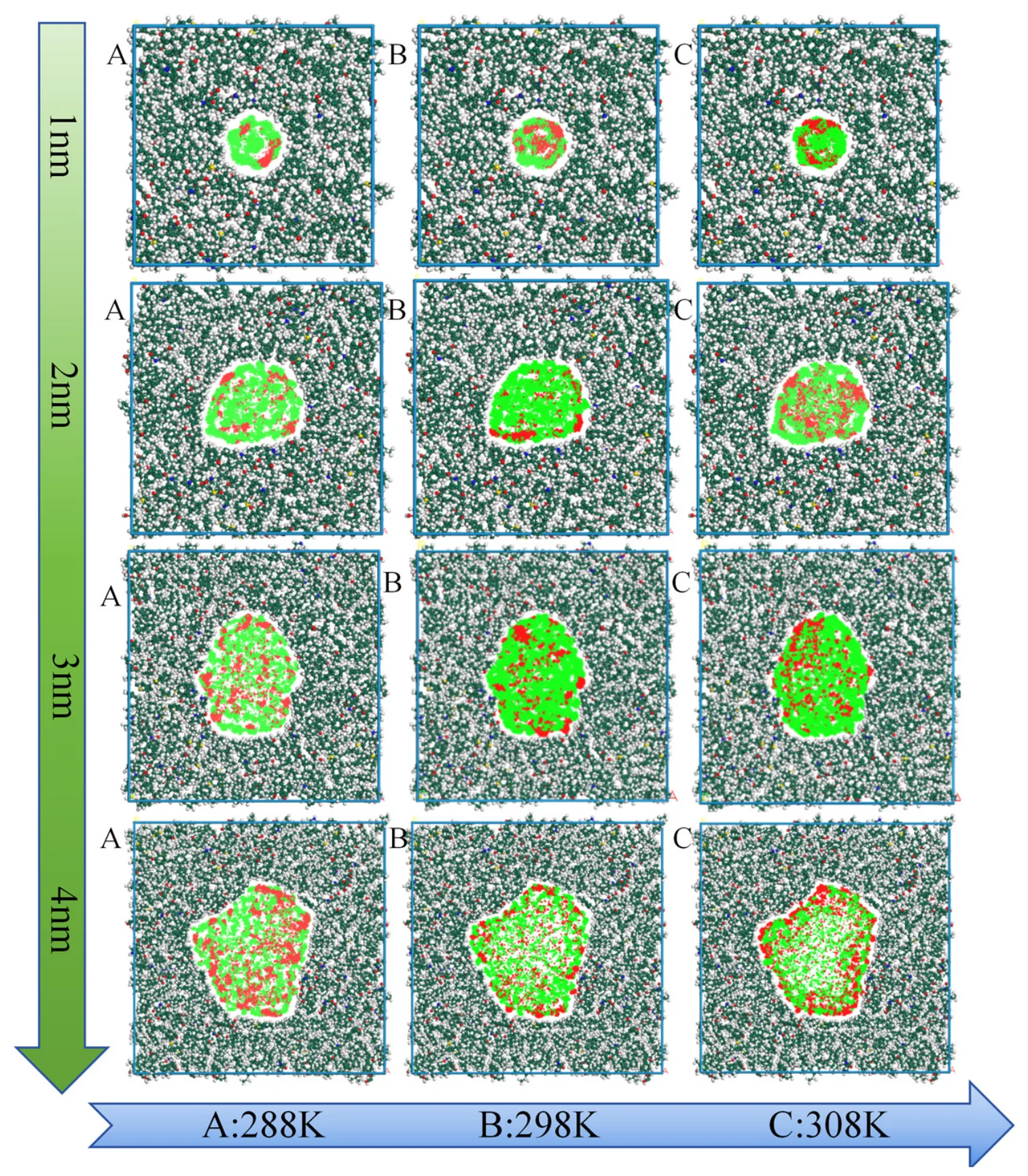
Spatial distributions of CH_4_/CO_2_ competitive adsorption in organic-matter pores at 8 MPa. The four rows (top to bottom) represent pore diameters of 1, 2, 3, and 4 nm, respectively, while columns (**A**–**C**) correspond to 288, 298, and 308 K, respectively. Red and green spheres represent adsorbed CH_4_ and CO_2_ molecules, respectively.

In the micropore range, the adsorption capacity of CO_2_ was much higher than that of CH_4_, and the increase in CH_4_ adsorption with pressure was very limited, indicating that CO_2_ exerts a strong inhibitory effect on CH_4_ adsorption. As the pore size increased, the adsorption amount of CO_2_ gradually decreased, whereas that of CH_4_ showed an increasing trend, and the difference between the adsorption capacities of the two gases gradually narrowed. This may be attributed to the fact that, with increasing pore size, more adsorption sites become exposed on the organic-matter pore surface. Although CO_2_ still exhibits preferential adsorption, the greater accessible contact space under conditions of declining total adsorption enhances the collision probability of CH_4_ molecules with the pore surface, thereby relatively strengthening CH_4_ adsorption. The competitive adsorption cloud maps of CH_4_ and CO_2_ in the organic-matter pores at 8 MPa are shown in Figure 13.

**Figure 14:**
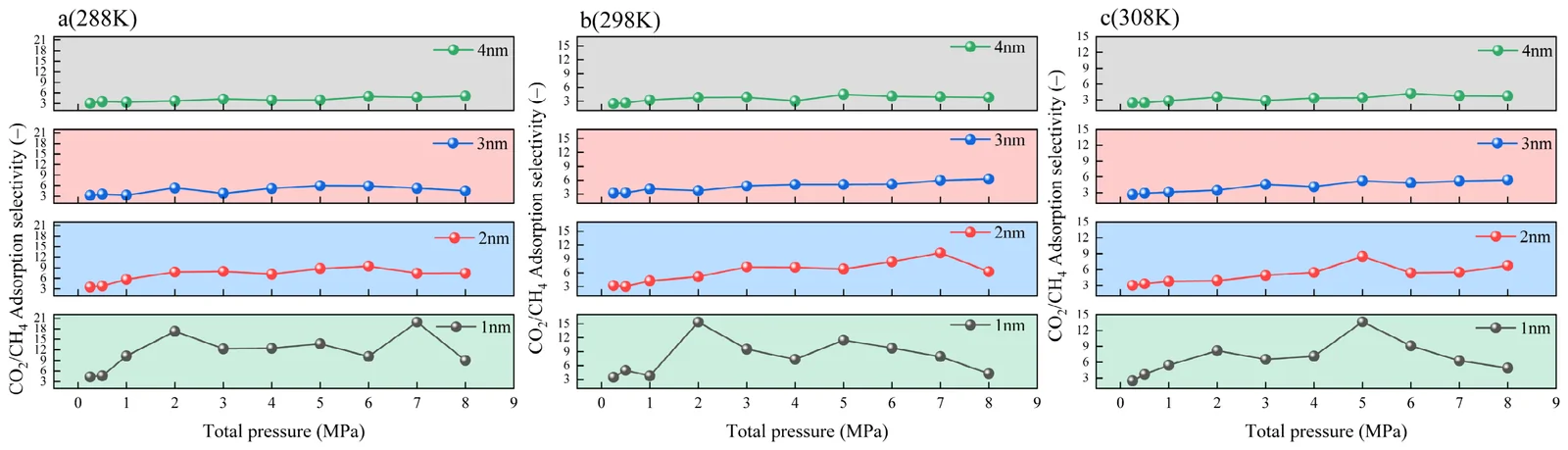
CO_2_/CH_4_ adsorption selectivity as a function of total pressure in the 1–4 nm organic-matter pore models at (**a**) 288 K, (**b**) 298 K, and (**c**) 308 K.

It can therefore be concluded that, under single-component adsorption conditions, CH_4_ exhibits the strongest adsorption capacity in the 1 nm organic-matter pore, whereas CO_2_ adsorption is strongest in the 2 nm pore. Under competitive adsorption conditions, the amount of CO_2_ adsorbed in micropores is substantially greater than that of CH_4_, while CH_4_ adsorption increases only slightly with increasing pressure. As the pore size increases, CO_2_ adsorption gradually decreases, whereas CH_4_ adsorption shows an increasing trend, resulting in a progressive narrowing of the difference between the adsorption capacities of the two gases. To quantify the competitive adsorption preference, the CO_2_/CH_4_ adsorption selectivity was calculated from the mean adsorbed molecule counts obtained in the binary GCMC simulations, using the definition provided in Section 3.3. Figure 14 presents the resulting selectivity as a function of pressure for the four nominal pore diameters at 288, 298, and 308 K. This comparison allows the effects of pore size, pressure, and temperature on the equilibrium adsorption selectivity to be evaluated directly. Because the component adsorption amounts are obtained from the same pore configurations, the common nominal-pore-volume normalization cancels when their ratio is calculated. Nevertheless, the resulting selectivity remains dependent on the structural characteristics of the selected pore models.

### 4.4. Pore-Size-Dependent Competitive CH_4_/CO_2_ Adsorption in Short-Chain-Aromatic Organic-Matter Pores

HRTEM characterization showed that the organic matter in the coal-measure shale is dominated by short-chain aromatic units, including benzene-, naphthalene-, and anthracene-like structures, and is characterized by a low degree of aromatic condensation and limited structural ordering. These characteristics indicate that the organic-matter pore walls are not composed of highly ordered, continuous, and planar large aromatic sheets. Instead, they constitute heterogeneous adsorption interfaces formed by short-chain aromatic fragments and their edge structures. Consequently, the adsorption of CH_4_ and CO_2_ in organic-matter nanopores is controlled not only by pore size but also by the aromatic structure of the pore walls, the distribution of local adsorption sites, and spatial confinement within the pores. Within the present model series, the short-chain aromatic fragments provide a common pore-wall structural framework, whereas the simulated adsorption differences across 1–4 nm pores are consistent with pore-size-dependent variations in molecular confinement and accessible adsorption space.

The single-component adsorption results demonstrate that CH_4_ and CO_2_ respond differently to variations in pore size. CH_4_ exhibited the highest adsorption capacity per unit pore volume in the 1 nm pore, indicating that its adsorption is governed predominantly by the micropore-filling mechanism. In the 1 nm pore, the pore size approaches the molecular dimensions of the adsorbate, allowing the potential fields generated by opposing pore walls to overlap. CH_4_ molecules are therefore subjected to stronger confinement, which enhances their adsorption capacity per unit pore volume. As the pore size increased from 1 to 4 nm, the pore volume increased, but the influence of the pore walls per unit volume gradually weakened, resulting in a decrease in the volumetric adsorption capacity of CH_4_. By contrast, within the present model series and under the adopted nominal-pore-volume normalization, CO_2_ exhibited its highest volumetric adsorption in the 2–3 nm pores rather than the 1 nm pore. This finding indicates that CO_2_ adsorption depends not only on the strength of pore-wall interactions but also on molecular accessibility and the available configurational space within the pores. Excessive spatial confinement in the 1 nm pore may restrict the entry and distribution of CO_2_ molecules, whereas the 2–3 nm pores provide both sufficiently strong pore-wall interactions and adequate adsorption space, thereby favoring CO_2_ enrichment. Accordingly, CH_4_ exhibited its highest volumetric adsorption capacity in the 1 nm pore, whereas that of CO_2_ was highest in the 2–3 nm pores.

In the binary CH_4_/CO_2_ system, CO_2_ exhibited a markedly stronger competitive adsorption ability than CH_4_. Under mixed-gas conditions, the adsorption capacities of both gases were lower than their corresponding single-component values, confirming competition for adsorption sites. The decrease in CH_4_ adsorption was considerably greater than that in CO_2_ adsorption, indicating that CO_2_ preferentially occupies energetically favorable adsorption sites and competitively excludes or suppresses CH_4_. This behavior is associated with the smaller molecular size of CO_2_ and its stronger interactions with the pore walls. Within confined pore spaces, CO_2_ can enter micropores more readily and preferentially occupy strong adsorption sites, thereby reducing the opportunities for CH_4_ molecules to contact and adsorb onto the pore walls. This effect is particularly pronounced in 1–2 nm pores, where the available space is limited and competition for adsorption sites is more intense. The preferential occupation of adsorption sites by CO_2_ is therefore amplified, leading to substantial suppression of CH_4_ adsorption. Consistent with this interpretation, the adsorption capacity of CO_2_ in micropores was much greater than that of CH_4_, and CO_2_ exerted a strong inhibitory effect on CH_4_ adsorption [52].

As the pore size increased to 3–4 nm, the competitive advantage of CO_2_ over CH_4_ gradually weakened. On the one hand, the larger pore space increased the opportunities for CH_4_ molecules to collide and interact with the pore walls. On the other hand, the enlargement of the pores weakened the overlap of the potential fields generated by opposing pore walls, thereby reducing the preferential occupation of the limited high-energy adsorption sites by CO_2_. Consequently, although CO_2_ retained preferential adsorption in the 3–4 nm pores, its inhibitory effect on CH_4_ was less pronounced than that in the 1–2 nm pores. The relative adsorption capacity of CH_4_ therefore increased, and the difference between the adsorption capacities of the two gases gradually narrowed. These results demonstrate that the competitive advantage of CO_2_ is not constant but strongly dependent on pore size: CO_2_ exhibits a stronger competitive adsorption advantage in the smaller 1 and 2 nm pore models, whereas the relative adsorption ability of CH_4_ increases in the larger 3 and 4 nm mesopores. This interpretation is consistent with the competitive adsorption results, which showed a gradual decrease in CO_2_ adsorption and an increase in CH_4_ adsorption with increasing pore size, accompanied by a progressive narrowing of the difference between the two gases.

Overall, the short-chain aromatic structure of coal-measure shale organic matter does not independently or directly determine competitive CH_4_/CO_2_ adsorption; rather, it exerts its regulatory effect through pore-size-dependent mechanisms. Short-chain aromatic structures provide the pore-wall framework characterized by low condensation, limited ordering, and localized aromatic adsorption sites, whereas variations in pore size from 1 to 4 nm further modify the overlap of pore-wall potential fields, the space accessible to gas molecules, and the competition for adsorption sites. For CH_4_, strong confinement in 1 nm micropores favors micropore filling. For CO_2_, 2–3 nm pores provide a more favorable adsorption space. In the binary system, 1–2 nm pores intensify the competitive suppression of CH_4_ by CO_2_, whereas the weaker spatial confinement in 3–4 nm pores enhances the relative adsorption ability of CH_4_. Therefore, the observed adsorption trends primarily characterize pore-size-dependent confinement within the selected HRTEM-informed organic-matter models. Quantifying the independent contribution of aromatic condensation or ordering would require controlled comparisons with alternative pore-wall structures.

Published experimental adsorption studies have reported higher single-component CO_2_ uptake than CH_4_ and preferential CO_2_ adsorption in CH_4_/CO_2_ mixtures [35,36], consistent with the general adsorption trends obtained here. In addition, the experimentally characterized precursor organic-matter model showed close agreement between calculated and measured helium densities and comparable CO_2_-probe micropore volumes [38]. These comparisons support the physical plausibility of the adopted structural framework and the qualitative adsorption trends. However, because experimental shale specimens and the present idealized organic-matter pores differ in composition and pore structure, the adsorption maxima observed in the nominal 2–3 nm pores are interpreted as characteristics of the present model series rather than universally optimal pore sizes. It should also be emphasized that the comparisons with published adsorption experiments and the experimentally characterized precursor molecular model do not constitute direct quantitative validation of the CO_2_/CH_4_ selectivity predicted for the present 1–4 nm pore models. Furthermore, one representative annealed pore configuration was adopted for each nominal pore diameter, and the effects of independently generated pore-wall configurations were not evaluated. Therefore, the calculated selectivity values should be regarded as equilibrium predictions for the selected molecular structures, without quantified uncertainty arising from structural realizations.

The preferential adsorption of CO_2_ over CH_4_ observed in the present models reflects equilibrium competition within dry organic-matter nanopores rather than directly demonstrating dynamic CH_4_ displacement. In actual coal-measure shale, moisture may occupy polar adsorption sites and reduce accessible pore space, while clay minerals and other inorganic constituents provide additional adsorption interfaces that can modify competitive adsorption. Furthermore, gas transport through connected pore networks, adsorption-induced swelling, and reservoir-scale flow behavior are important factors governing the effectiveness of CO_2_ injection. Therefore, the present findings provide molecular-scale insights relevant to the evaluation of CO_2_-enhanced gas recovery and geological storage, while quantitative assessments of displacement efficiency and storage performance require consideration of these additional processes [40].

An important limitation of the present study is that the GCMC simulations were conducted using pre-optimized but rigid organic-matter pore structures. Under high-pressure CO_2_ adsorption conditions, organic matter may undergo adsorption-induced swelling and local rearrangement of its molecular framework, while the surrounding matrix may also experience mechanical compression under reservoir stresses. These coupled effects can modify the effective pore dimensions, accessible pore volume, and distribution of energetically favorable adsorption sites. Local pore narrowing may restrict gas accessibility, whereas structural relaxation or pore opening may provide additional adsorption space. Consequently, the adsorption capacities predicted using rigid pore structures may either underestimate or overestimate those of deformable systems, depending on the resulting pore geometry and the definition of adsorption capacity. Furthermore, because CO_2_ and CH_4_ interact differently with the organic framework, deformation-induced changes in pore accessibility and adsorption-site environments may alter their competitive adsorption behavior and CO_2_/CH_4_ selectivity. These effects could be particularly significant in 1–2 nm pores, where small structural variations can substantially modify molecular confinement. Previous molecular simulations have demonstrated that adsorption-induced swelling and kerogen flexibility can influence gas uptake and competitive selectivity [53,54]. Therefore, the pore-size-dependent adsorption capacities and preferential CO_2_ adsorption reported in this study should be interpreted within the fixed-framework approximation. Future investigations combining GCMC with flexible-framework molecular dynamics simulations under representative confining stresses are needed to quantify adsorption–deformation coupling and assess its effects on the predicted pore-size dependence.

Li et al. [55] investigated CH_4_/C_2_H_6_ sorption in kerogen nanopore systems containing micropores and mesopores, highlighting the importance of distinguishing their sorption contributions and using appropriate normalization bases when comparing molecular simulations with experimental isotherms. Although their gas mixture and pore models differ from those considered here, this methodological perspective emphasizes the need to quantify probe-accessible pore volumes and surface areas after annealing and to assess the sensitivity of the present adsorption maxima to alternative normalization methods.

## 5. Conclusions

The organic matter in the coal-measure shale of the Qinshui Basin exhibits a typical “short-chain aromatic” molecular structure. Statistical analysis of HRTEM lattice fringes showed that the number ratio of 1 × 1, 2 × 2, and 3 × 3 aromatic units was approximately 8:4:3, collectively accounting for more than 90% of the identified fringes. The number of fused aromatic rings was mainly concentrated between two and four, and the lattice fringes were generally short. A dominant orientation of 150–160° was also observed, indicating a preferred alignment of the aromatic structures. These characteristics demonstrate that the organic matter has a relatively low degree of aromatic condensation and limited structural ordering. These structural characteristics provide the experimental basis for the short-chain aromatic pore-wall models used to investigate pore-size-dependent CH_4_/CO_2_ competitive adsorption.

The single-component adsorption of CH_4_ and CO_2_ exhibited distinctly different pore-size-dependent patterns. CH_4_ showed the highest adsorption capacity per unit pore volume in the 1 nm pore, followed by a gradual decrease with increasing pore size, indicating that CH_4_ adsorption is governed predominantly by the micropore-filling mechanism. By contrast, the volumetric adsorption capacity of CO_2_ initially increased and then decreased with increasing pore size, reaching its maximum in the 2–3 nm pores. This result suggests that CO_2_ adsorption is controlled not only by pore-wall interactions but also by molecular accessibility and the number of available adsorption sites.

Significant competitive adsorption occurred in the binary CH_4_/CO_2_ system, with CO_2_ exhibiting a stronger competitive adsorption ability than CH_4_. The adsorption capacities of both gases in the mixed system were lower than their corresponding single-component values. The reduction in CH_4_ adsorption was considerably greater than that in CO_2_ adsorption, indicating that CO_2_ preferentially occupies energetically favorable adsorption sites and consequently suppresses CH_4_ adsorption.

The competitive advantage of CO_2_ over CH_4_ exhibited a pronounced dependence on pore size. In the nominal 1 nm micropore and 2 nm mesopore, the relatively limited pore space favored preferential CO_2_ adsorption, resulting in pronounced competitive suppression of CH_4_ adsorption. As the pore size increased to 3–4 nm, the available pore space increased and CH_4_ molecules gained more opportunities to interact with the pore walls. Consequently, the competitive advantage of CO_2_ gradually weakened, and the difference between the adsorption capacities of CH_4_ and CO_2_ decreased.

Within the HRTEM-informed short-chain aromatic pore models, competitive CH_4_/CO_2_ adsorption exhibited systematic pore-size dependence, reflecting the influence of nanoscale confinement and accessible adsorption space. These equilibrium adsorption trends provide molecular-scale insights relevant to CO_2_-enhanced gas recovery but do not directly quantify CH_4_ displacement efficiency, reservoir-scale recovery factors, or geological CO_2_ storage capacity. However, because models with different aromatic condensation degrees or structural ordering were not included, the independent influence of short-chain aromaticity remains to be established through controlled comparisons.

It should be noted that these conclusions are based on pre-optimized, rigid organic-matter pore structures. Adsorption-induced swelling and stress-dependent framework deformation may modify the quantitative adsorption capacities and CO_2_/CH_4_ selectivity, particularly in narrow nanopores, and should be further investigated using coupled adsorption–deformation simulations. Furthermore, the CO_2_ adsorption maximum observed in the nominal 2–3 nm pores and the combined CH_4_/CO_2_ adsorption maximum in the nominal 2 nm pore are model-dependent results obtained using nominal-pore-volume normalization. Because the probe-accessible pore volumes and surface areas of the annealed structures were not quantified, these pore sizes cannot be regarded as universally optimal without further evaluation of pore accessibility and normalization sensitivity. The quantitative CO_2_/CH_4_ selectivity trends also remain subject to uncertainty because direct validation against corresponding binary-gas adsorption experiments and assessments using independent pore-wall realizations have not yet been performed.

## Figures and Tables

**Table 1:** The size distribution range of aromatic structures.

Aromatic Structure	MinL (nm)	MaxL (nm)	Mean d/nm	d/nm
1 × 1	0.28	0.49	0.39	0.30–0.54
2 × 2	0.49	0.71	0.60	0.55–0.74
3 × 3	0.74	1.13	0.93	0.75–1.14
4 × 4	0.98	1.56	1.27	1.15–1.44
5 × 5	1.23	1.98	1.60	1.45–1.74
6 × 6	1.47	2.41	1.94	1.75–2.04
7 × 7	1.72	2.84	2.28	2.05–2.44
8 × 8	1.96	3.26	2.61	2.45–2.84

**Table 2:** Statistical results of aromatic lattice-fringe lengths in five selected HRTEM image regions (Figure 4A–E).

Aromatic Ring Type		1 × 1	2 × 2	3 × 3	4 × 4	5 × 5	6 × 6	7 × 7	8 × 8
Image A	Number	359	153	111	34	11	5	4	1
	Proportion	52.9%	22.6%	16.4%	5.0%	1.6%	0.7%	0.6%	0.1%
Image B	Number	254	187	117	34	9	6	4	1
	Proportion	41.5%	30.6%	19.1%	5.6%	1.5%	1.0%	0.7%	0.2%
Image C	Number	308	136	120	33	18	15	5	3
	Proportion	48.3%	21.3%	18.8%	5.2%	2.8%	2.4%	0.8%	0.5%
Image D	Number	350	183	120	22	8	3	2	0
	Proportion	50.9%	26.6%	17.4%	3.2%	1.2%	0.4%	0.3%	0.0%
Image E	Number	326	138	135	42	20	9	9	2
	Proportion	47.9%	20.3%	19.8%	6.2%	2.9%	1.3%	1.3%	0.3%
Total	Number	1597	797	603	165	66	38	24	7
	Proportion	48.4%	24.2%	18.3%	5.0%	2.0%	1.2%	0.7%	0.2%

## Data Availability

The data that support the findings of this study are available from the corresponding author upon reasonable request.

## Supplementary Materials

The following supporting information can be downloaded at: https://www.mdpi.com/article/10.3390/molecules31214038/s1. Detailed Molecular Modeling and GCMC Simulation Procedures: Section S1: Preparation and Geometry Optimization of Adsorbate Molecules; Section S2: Construction and Geometry Optimization of Organic-Matter Pore Models; Section S3: Molecular Dynamics Annealing of Organic-Matter Pore Structures; Section S4: Grand Canonical Monte Carlo Adsorption Simulations; Section S5: Adsorption Selectivity and Data Processing.
