# Supplementary material for: Pore-Size-Dependent Competitive CH4/CO2 Adsorption in HRTEM-Informed Short-Chain-Aromatic Organic-Matter Nanopores of Coal-Measure Shale
Source: Molecules. 2026 Nov 9;31(21):4038. doi: 10.3390/molecules31214038 (PMC13764924; doi:10.3390/molecules31214038)
Supplement: Supplementary file 1 [file molecules-31-04038-s001.zip › molecules-4627219-supplementary.pdf]

# Pore-Size-Dependent Competitive $CH_4$/$CO_2$ Adsorption in HRTEM-Informed Short-Chain-Aromatic Organic-Matter Nanopores of Coal-Measure Shale

## Supplementary Materials

**Detailed Molecular Modeling and GCMC Simulation Procedures**

S1. Preparation and Geometry Optimization of Adsorbate Molecules

The $CH_4$ and $CO_2$ molecular structures used in the adsorption calculations were prepared using the molecular mechanics capabilities of Materials Studio. The DREIDING force field was selected for the molecular preparation procedure.

In the Energy settings, the calculation quality was set to Medium, and the existing atomic charges were retained. Both electrostatic and van der Waals interactions were evaluated using the atom-based summation method. A cubic-spline cutoff scheme was employed for nonbonded interactions, with a cutoff distance of 12.5 Å, a spline width of 1 Å, and a buffer width of 0.5 Å.

Molecular geometry optimization was performed using the Geometry Optimization task and the Smart algorithm. The convergence quality was set to Medium. The energy and force convergence thresholds were specified as 0.001 kcal/mol and 0.5 kcal/mol/Å, respectively, and the maximum number of optimization iterations was 500.

The optimized $CH_4$ and $CO_2$ molecular structures were subsequently used as adsorbate molecules in the Sorption calculations. Their molecular geometries were treated as rigid during the GCMC adsorption simulations.

S2. Construction and Geometry Optimization of Organic-Matter Pore Models

The organic-matter pore models were constructed using molecular fragments derived from the experimentally characterized precursor molecular structure $C_{177}H_{160}N_2O_8S$. The aromatic structural characteristics obtained from HRTEM lattice-fringe analysis were incorporated into the molecular modeling procedure.

The dominant $1 \times 1$, $2 \times 2$, and $3 \times 3$ aromatic structural units were represented according to their approximate relative abundance ratio of 8:4:3. The molecular fragments were assembled around nominally cylindrical pore spaces to construct four organic-matter pore models with diameters of 1, 2, 3, and 4 nm.

The corresponding simulation-cell dimensions were $5 \times 5 \times 1.5$, $6 \times 6 \times 1.5$, $7 \times 7 \times 1.5$, and $8 \times 8 \times 1.5$ $nm^3$, respectively. The numbers of organic-matter molecular fragments used for the four pore models were 12, 15, 21, and 26, respectively. The target density of the organic-matter framework was

1.40 g/cm$^3$ .

Following model construction, molecular mechanics geometry optimization was conducted using the Geometry Optimization task in the Forcite module of Materials Studio. The DREIDING force field and Smart optimization algorithm were employed. The convergence quality was set to Fine, and the maximum number of optimization iterations was 1,000.

Both electrostatic and van der Waals interactions were calculated using the atom-based summation method, with a cutoff distance of 12.5 Å.

This optimization procedure was applied to the assembled organic-matter pore frameworks, separately from the preparation of the individual $CH_4$ and $CO_2$ adsorbate molecules described in Section S1.

## S3. Molecular Dynamics Annealing of Organic-Matter Pore Structures

After geometry optimization, molecular dynamics annealing was performed using the Anneal task in the Forcite module to obtain representative low-energy configurations of the organic-matter pore structures.

The annealing simulations were conducted in the canonical ensemble (NVT) using the DREIDING force field. Both electrostatic and van der Waals interactions were evaluated using the atom-based summation method, consistent with the preceding pore-framework optimization procedure.

The temperature was increased from 300 K to 600 K in increments of 60 K and subsequently decreased to 300 K at the same rate. The total annealing simulation time was 200 ps, with a molecular dynamics time step of 1 fs. Temperature was regulated using the Nosé thermostat, and 10 annealing cycles were performed.

At the end of each annealing cycle, the resulting molecular configuration was subjected to further molecular mechanics optimization to obtain a low-energy structure. The energy evolution of the annealed configurations was examined, and the configuration with the lowest potential energy was selected as the representative pore structure for subsequent adsorption calculations.

During geometry optimization and annealing, the initially cylindrical pores with diameters of 2 – 4 nm underwent slight structural relaxation, resulting in elliptical or irregularly rounded pore geometries.

The selected low-energy configurations were subsequently used as fixed organic-matter frameworks during GCMC adsorption calculations. Thus, structural relaxation was performed during pore-model preparation, whereas the atomic positions of the pore walls remained fixed during adsorption sampling.

## S4. Grand Canonical Monte Carlo Adsorption Simulations

Single-component adsorption and binary competitive adsorption simulations of $CH_4$ and $CO_2$ were conducted using the Sorption module of Materials Studio under periodic boundary conditions.

The calculation task was specified as Fixed Pressure, the Monte Carlo method was Metropolis, and Customized task settings were employed.

The DREIDING force field was used to describe the molecular interactions. The partial atomic charges already assigned to the molecular structures were retained during the adsorption calculations.

Electrostatic interactions were evaluated using Ewald summation, whereas van der Waals interactions were calculated using the atom-based summation method. A cutoff distance of 12.5 Å and a cubic-spline cutoff scheme were adopted for the van der Waals interactions.

The organic-matter pore walls and adsorbate molecules were treated as rigid during the GCMC calculations. Therefore, the adsorption simulations were performed within the optimized pore configurations obtained after geometry optimization and annealing.

S4.1. Gas Composition and Thermodynamic Conditions

The adsorption simulations were performed at temperatures of 288, 298, and 308 K.

The investigated nominal total-pressure points were 0.25, 0.50, 1, 2, 3, 4, 5, 6, 7, and 8 MPa.

For the binary competitive adsorption simulations, $CH_4$ and $CO_2$ were introduced at a fixed molar mixing ratio of 1:1. Accordingly, the bulk-gas mole fraction of each component was 0.5:

$y(CH_4) = y(CO_2) = 0.5$.

The nominal partial pressure of each component was defined as:

$p_i = y_iP$,

where $p_i$ represents the nominal partial pressure of component i, $y_i$ denotes its bulk-gas mole fraction, and P is the total pressure.

For the equimolar $CH_4/CO_2$ mixture, the nominal partial pressures of the two components were therefore equal, with each accounting for one-half of the total pressure.

The same gas composition was maintained for all four pore models and all investigated temperature and pressure conditions.

The recorded Sorption calculation settings included fugacity inputs of 250, 500, 1,000, 2,000, 3,000, 4,000, 5,000, 6,000, 7,000, and 8,000 kPa. The nominal pressure conditions and the recorded fugacity inputs are specified separately to describe the thermodynamic settings used in the adsorption calculations.

S4.2. GCMC Sampling and Computational Settings

Each GCMC adsorption calculation consisted of an equilibration stage followed by a production stage.

The equilibration stage comprised 200,000 Monte Carlo steps, followed by 400,000 production steps for statistical sampling.

The equilibration stage was used to establish the adsorption configuration under the prescribed thermodynamic conditions. The production-stage sampling results were used to determine the mean numbers of adsorbed $CH_4$ and $CO_2$ molecules.

The Monte Carlo moves included exchange, conformational changes, rotation, translation, and regrowth.

The requested simulation outputs included energy distributions, density fields, and energy fields.

The field-sampling interval was set to 25 steps. The grid resolution was specified as Medium, with a grid spacing of 0.4 Å.

The same computational procedure was applied consistently to the four representative organic-matter pore models.

S5. Adsorption Selectivity and Data Processing

The preferential adsorption of $CO_2$ relative to $CH_4$ in the binary mixture was quantified using the adsorption selectivity, defined as the ratio of the adsorbed-phase composition ratio to the corresponding bulk-gas composition ratio:

$S(CO_2/CH_4) = [x(CO_2)/x(CH_4)] / [y(CO_2)/y(CH_4)]$,

where x and y denote the mole fractions of the corresponding gas components in the adsorbed phase and bulk-gas phase, respectively.

Because the binary $CH_4/CO_2$ mixture was specified at a fixed molar ratio of 1:1, the bulk-gas composition ratio was unity. Consequently, the selectivity was calculated directly from the mean adsorbed molecule counts of $CO_2$ and $CH_4$:

$S(CO_2/CH_4) = \langle N(CO2) \rangle / \langle N(CH_4) \rangle$,

where $\langle N(CO_2) \rangle$ and $\langle N(CH_4) \rangle$ represent the mean adsorbed molecule counts obtained from the same binary GCMC simulation.

The adsorption selectivity was calculated separately for each nominal pore diameter, temperature, and pressure condition.

The volumetric adsorption quantities were calculated using the nominal cylindrical pore volumes as the normalization basis. Because the same pore-volume normalization factor was applied to both components in each binary adsorption calculation, this common factor cancels when the component adsorption ratio is used to calculate selectivity.

Accordingly, the $CO_2/CH_4$ adsorption selectivity is a dimensionless quantity characterizing the competitive adsorption preference under the specified molecular simulation conditions.
